# Design and statistical optimisation of emulsomal nanoparticles for improved anti-SARS-CoV-2 activity of *N*-(5-nitrothiazol-2-yl)-carboxamido candidates: *in vitro* and *in silico* studies

**DOI:** 10.1080/14756366.2023.2202357

**Published:** 2023-04-24

**Authors:** Ahmed A. Al-Karmalawy, Dalia S. El-Gamil, Rabeh El-Shesheny, Marwa Sharaky, Radwan Alnajjar, Omnia Kutkat, Yassmin Moatasim, Mohamed Elagawany, Sara T. Al-Rashood, Faizah A. Binjubair, Wagdy M. Eldehna, Ayman M. Noreddin, Mohamed Y. Zakaria

**Affiliations:** a Pharmaceutical Chemistry Department, Faculty of Pharmacy, Ahram Canadian University, Giza, Egypt; b Water Pollution Research Department, The Center of Scientific Excellence for Influenza Viruses, Environmental Research Institute, National Research Centre, Giza, Egypt; c Cancer Biology Department, Pharmacology Unit, National Cancer Institute (NCI), Cairo University, Cairo, Egypt; d Department of Chemistry, Faculty of Science, University of Benghazi, Benghazi, Libya; e Faculty of Pharmacy, Libyan International Medical University, Benghazi, Libya; f Department of Chemistry, University of Cape Town, Rondebosch, South Africa; g Department of Pharmaceutical Chemistry, Faculty of Pharmacy, Damanhour University, Damanhour, Egypt; h Department of Pharmaceutical Chemistry, College of Pharmacy, King Saud University, Riyadh, Saudi Arabia; i Department of Pharmaceutical Chemistry, Faculty of Pharmacy, Kafrelsheikh University, Kafrelsheikh, Egypt; j School of Biotechnology, Badr University in Cairo, Badr City, Egypt; k Department of Internal Medicine, School of Medicine, University of California Irvine, Irvine, CA, USA; l Department of Clinical Pharmacy, Faculty of Pharmacy, Ahram Canadian University, Giza, Egypt; m Department of Pharmaceutics and Industrial Pharmacy, Faculty of Pharmacy, Port Said University, Port Said, Egypt

**Keywords:** *N*-(5-nitrothiazol-2-yl)-carboxamide, emulsomes, anti-SARS-CoV-2, mechanistic study, SAR

## Abstract

In this article, emulsomes (EMLs) were fabricated to encapsulate the *N*-(5-nitrothiazol-2-yl)-carboxamido derivatives (**3a**–**3g**) in an attempt to improve their biological availability and antiviral activity. Next, both cytotoxicity and anti-SARS-CoV-2 activities of the examined compounds loaded EMLs (**F3a**–**g**) were assessed in Vero E6 cells via MTT assay to calculate the CC_50_ and inhibitory concentration 50 (IC_50_) values. The most potent **3e**-loaded EMLs (**F3e**) elicited a selectivity index of 18 with an IC_50_ value of 0.73 μg/mL. Moreover, **F3e** was selected for further elucidation of a possible mode of action where the results showed that it exhibited a combination of virucidal (>90%), viral adsorption (>80%), and viral replication (>60%) inhibition. Besides, molecular docking and MD simulations towards the SARS-CoV-2 Mpro were performed. Finally, a structure–activity relationship (SAR) study focussed on studying the influence of altering the size, type, and flexibility of the α-substituent to the carboxamide in addition to compound contraction on SARS-CoV-2 activity.

**Highlights**
- Emulsomes (EMLs) were fabricated to encapsulate the *N*-(5-nitrothiazol-2-yl)-carboxamido derivatives (**3a**–**3g**).
- The most potent **3e**-loaded EMLs (**F3e**) showed an IC_50_ value of 0.73 μg/mL against SARS-CoV-2.
- F3e exhibited a combination of virucidal (>90%), viral adsorption (>80%), and viral replication (>60%) inhibition.
- Molecular docking, molecular dynamics (MD) simulations, and MM-GBSA calculations were performed.
- Structure–activity relationship (SAR) study was discussed to study the influence of altering the size, type, and flexibility of the α-substituent to the carboxamide on the anti-SARS-CoV-2 activity.

## GRAPHICAL ABSTRACT

**Figure:**
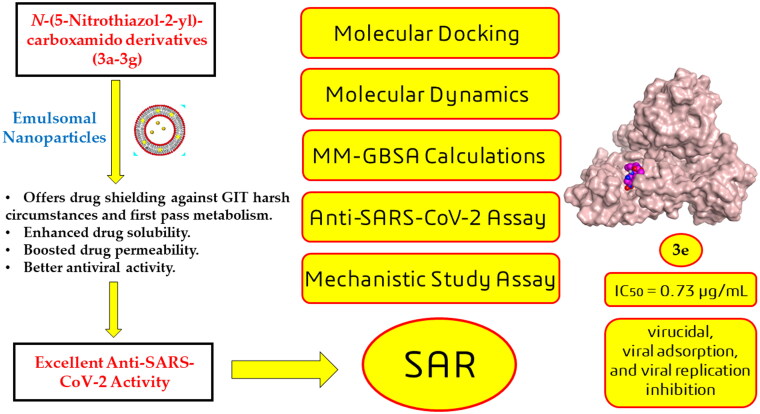

## Introduction

Since its emergence in late 2019, the COVID-19 pandemic (caused by the novel severe acute respiratory syndrome coronavirus 2 (SARS-CoV-2)) has gained global attention due to its significant mortality and morbidity with over 661 million confirmed cases and almost 6.7 million deaths (https://www.worldometers.info/coronavirus/)^1^. In the initial stages of the viral infection, symptoms are commonly mild and include fever, myalgia, and dry cough. At more advanced stages of the disease, pulmonary symptoms such as dyspnoea and hypoxia develop^2^. Although the viral load usually subsides by that time, the condition of some patients worsens due to an uncontrolled systemic inflammatory response (or a cytokine storm), resulting in long-term or life-threatening implications on lung tissues and other organs^3^.

Like SARS-CoV and Middle East respiratory syndrome coronavirus (MERS-CoV), SARS-CoV-2 belongs to the genus β-coronavirus and is an enveloped positive-stranded RNA virus. Its coronavirus particle consists of four structural proteins, namely the nucleocapsid, envelope, membrane, and spike^4^. In addition, its viral genome comprises non-structural open reading frames that encode several non-structural proteins, such as enzymes needed for viral intracellular replication^5^^,^^6^.

The entry of the SARS-CoV-2 into the host cell is mediated via the fusion of the viral spike (S) glycoprotein to cell surface angiotensinogen-converting enzyme 2 (ACE2) receptors. Subsequent conformational changes in the S protein promote the viral envelope fusion with the cell membrane through an endosomal pathway^7^. On entering the host cells, the viral genome is released as a positive single-stranded RNA that can be directly converted into viral polyproteins employing host cell protein translation machinery. These polyproteins are further hydrolysed by two viral proteases, namely the Mpro (also known as the 3-chymotrypsin-like protease (3CLpro)) and the papain-like protease (PLpro) to yield the viral structural and functional proteins, including the RNA-dependent RNA polymerase (RdRp)^5^. Afterwards, RdRp synthesises a full-length negative-strand RNA template, utilised to produce more viral genomic RNA (by replicating the new positive-strand RNA) in addition to several subgenomic RNAs, which are later translated into relevant viral proteins. Subsequently, viral proteins and RNA genomes are assembled into virions that are released from the cell via exocytosis^7^^,^^8^.

Despite the huge leap in the development of vaccines worldwide, the demand for pharmacological treatments of COVID-19 disease is still urging as vaccines were found not 100% effective, especially with emerging variants, and could not fully prevent the transmission of the disease besides their slow reach to underdeveloped countries^7^. On the contrary, pharmacological treatments can be designed to address multiple targets that are highly conserved among variants of the virus such as Mpro, PLpro, non-structural protein 12 (nsp12), and RdRp in addition to host-related targets^9^. This would likely maintain broad-spectrum therapeutic effectiveness despite the continuously emerging viral mutations.

Within the last three years, several antiviral therapeutic approaches have been tailored to various druggable targets along the stages of viral entry and replication. Recently, the orally active nirmatrelvir (PF-07321332) in combination with ritonavir, was granted emergency use by the FDA in December 2021 for COVID-19 treatment under the trade name Paxlovid^®^^10^. The combination elicited an efficacy of about 88% against hospitalisation or mortality in adult outpatients after its administration within five days of the onset of symptoms^11^. Nirmatrelvir is a peptidomimetic inhibitor of the main protease Mpro that acts via a reversible covalent interaction with its key Cys-145 residue^12^. Ritonavir lacks activity against SARS-CoV-2 Mpro but acts as a pharmacokinetic boosting agent by inhibiting the CYP3A-mediated metabolism of nirmatrelvir^13^. The most prominent antivirals against viral genome replication include the FDA-approved Remdesivir (Veklury^®^) and the EUA-authorised Molnupiravir (Lagevrio^®^)^14^.

Among all reported SARS-CoV-2 druggable targets, Mpro is not only the most cysteine protease conserved in structure but also its function in all known CoVs^15^. It serves as the main protease for proteolytic cleavage of the two overlapping large polyprotein precursors (pp1a and pp1ab) into the 16 non-structural proteins required for viral replication and maturation^5^. Furthermore, it is a key player in virus entry to host cells where its inhibition was reported to impede viral entry and the subsequent infection^16^. The substantial dependence of the virus on the proper functioning of this protease, along with the absence of a homologous human protease, makes Mpro one of the most pursued therapeutic targets for curbing coronavirus-associated diseases^17–19^.

**Figure 1.**
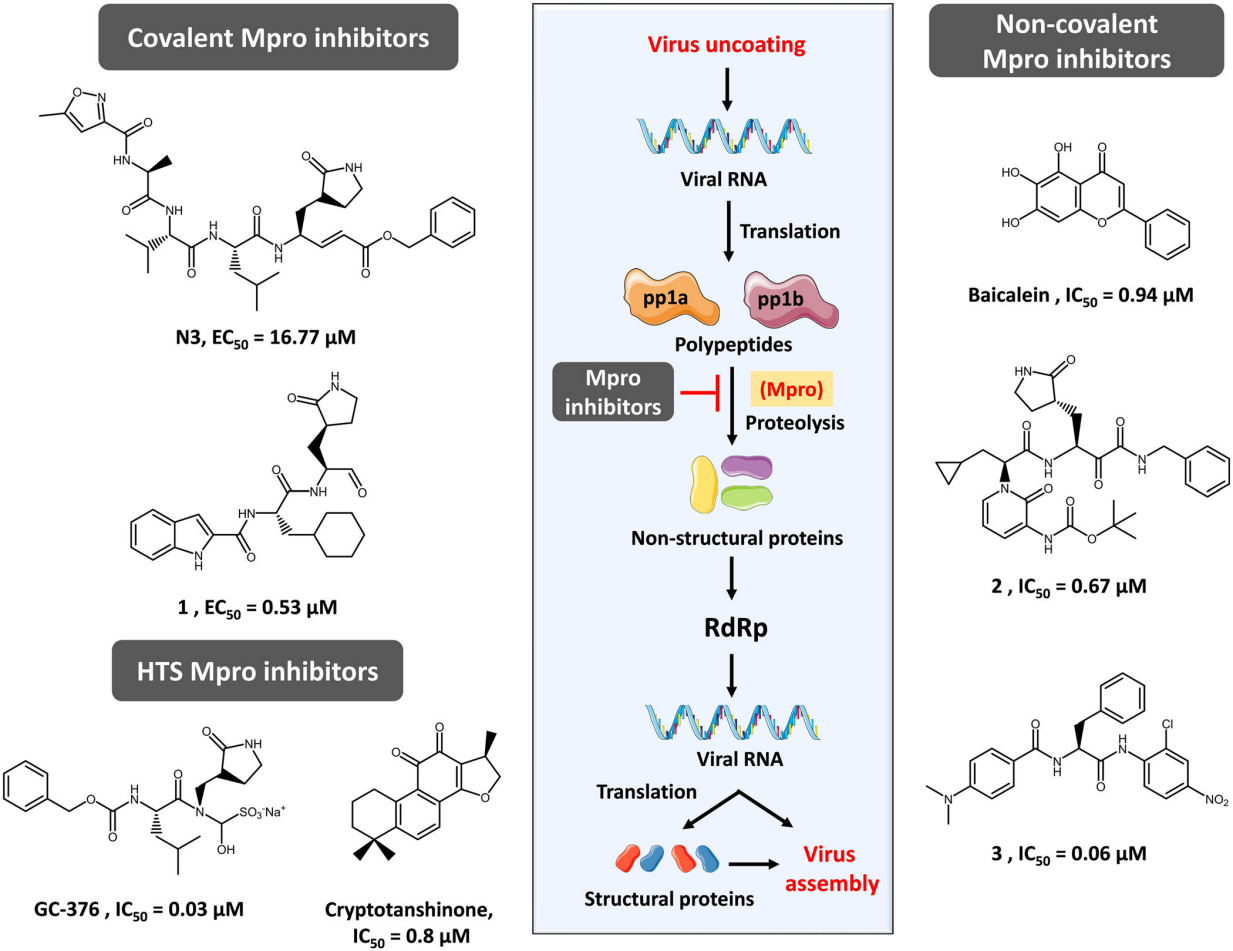
Examples of reported potential Mpro inhibitors and how they function against SARS-CoV-2.

Several studies, comprehensively reviewed in refs.^20–22^, have successfully disclosed various covalent (e.g. N3^23^ and 1^17^), non-covalent (e.g. baicalein^20^, 2^24^, and 3^25^) and high-throughput screening (HTS) (e.g. GC-376^26^ and cryptotanshinone^27^) potential SARS-CoV-2 Mpro inhibitors (Figure 1). Among these, α-ketoamide inhibitors, peptide-based inhibitors, anilide-based inhibitors, indole carboxamide-based inhibitors, drugs from Chinese traditional medicine, phytochemicals, and microorganism-based inhibitors were the most extensively explored drug classes^20^^,^^28^.

Diverse inhibitors exhibited potent inhibitory activities against SARS-CoV-2; however, the majority of drugs are suspended in preclinical experiments and terminated within *in vivo* models due to pharmacodynamics or pharmacokinetics concerns^21^. The main challenge in designing protease inhibitors is a dilemma between using highly potent and selective peptidomimetic/high molecular weight compounds but of poor drug-likeness versus using non-peptidomimetic/low molecular weight compounds of lower inhibitory potential^29^^,^^30^. Accordingly, continuous endeavours on SARS-CoV-2 Mpro inhibitors aim at achieving the required pharmacodynamic/kinetic balance that ensures clinical effectiveness and tolerability.

Among the most frequent reasons for poor absorption and restricted oral bioavailability of candidate drugs are their diminished intestinal permeability besides their liability for extensive metabolism in GIT and liver^31^. Accordingly, a lipidic vesicular system, i.e. liposome or noisome is usually adopted to overcome those pitfalls^32^. Emulsomes (EMLs) are one of the vesicular systems that possess the characteristics of lipidic bilayered vesicles and nanoemulsions and even exhibit the advantages of both^33^. Emulsomes primarily comprise two basic components: solid lipid as core enveloped with phospholipid as an outer layer which augments the vesicle stability. Emulsomes can successfully accommodate both hydrophilic and lipophilic drugs, in addition to overcoming the defects of conventional vesicular systems such as the high liability of aggregation and elevated drug leakage rate^34^. Moreover, surface PEGylation of EMLs could promote vesicle stability and prolong its duration in the systemic circulation^35^. Accordingly, EMLs are deemed as a prosperous carrier for candidate drugs, boosting their antiviral activity and bioavailability.

### The rationale for work design

**Scheme 1.**
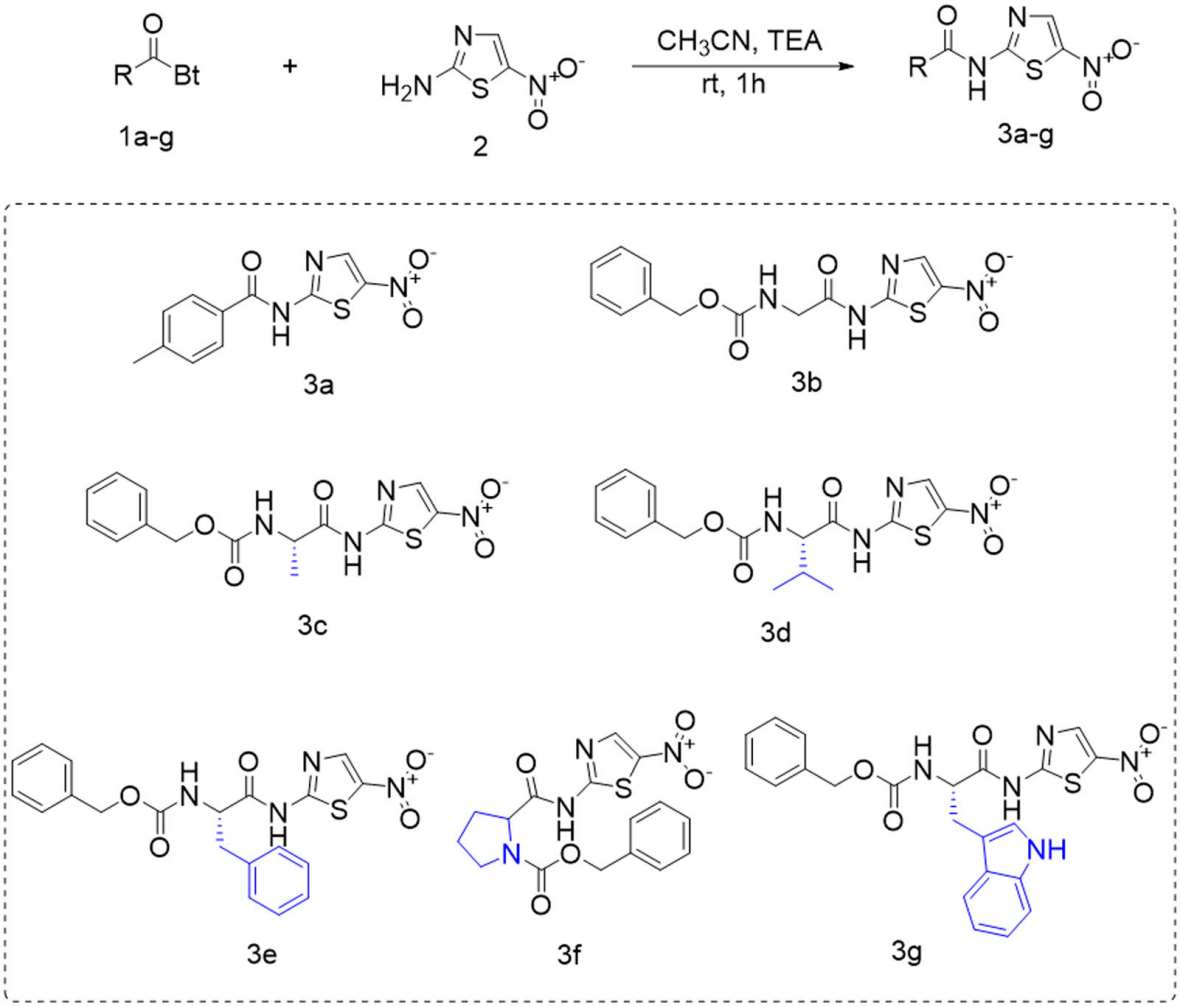
Chemical synthesis of the previously designed **3a**–**g** candidates to combat COVID-19^31^.

Previously, a series of *N*-(5-nitrothiazol-2-yl)-carboxamido derivatives (**3a**–**g**) was designed based on the basic pharmacophoric features of the co-crystallised inhibitor of the SARS-CoV. This was done according to the fact that there is a close structural similarity between the two strains of SARS-CoV (1 and 2)^31^. However, only compound **3b** (Scheme 1) showed superior anti-SARS-CoV-2 (174.7 µg/mL) and almost the SARS-CoV-2 Mpro inhibition (5.12 µg/mL) activities as well. This was attributed to its proposed better penetration throughout SARS-CoV-2 infected cells. Interestingly, one of the previous recommendations was to apply a suitable formulation for compound **3d** to explain its superior *in silico* stability in contrast to its lower anti-SARS-CoV-2 activity, compared to other members^31^. Herein, the proceeded study EMLs were fabricated to encapsulate the examined compounds (**3a**–**g**) in an attempt to improve their biological availability and antiviral activity as well.

**Figure 2.**
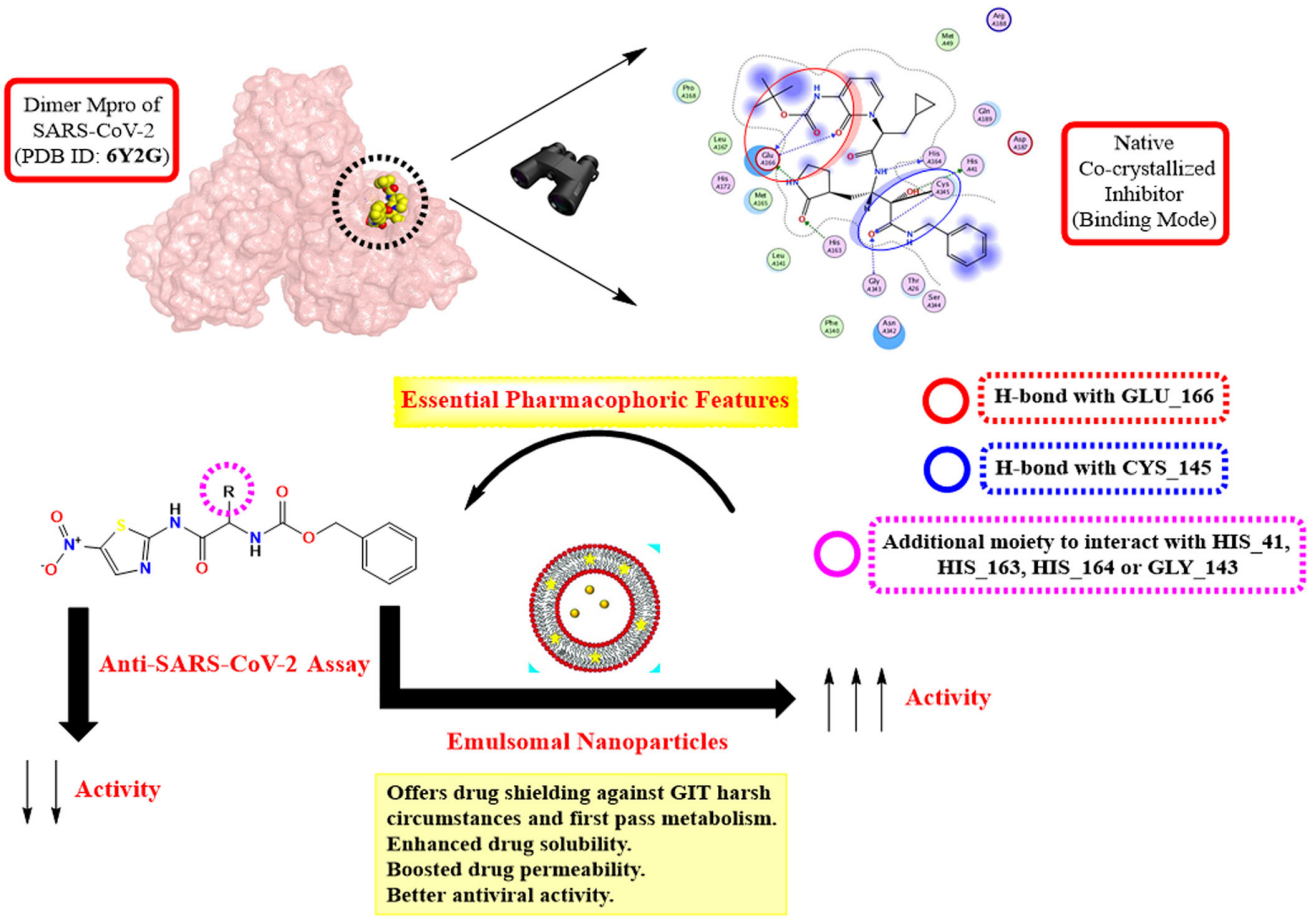
Rationale design for the synthesised *N*-(5-nitrothiazol-2-yl)-carboxamido derivatives as potential inhibitors of SARS-CoV-2 Mpro and the promising effect of the emulsomal nanoparticles.

EMLs aid in promoting intestinal permeability and prolonging the mean residence time of the drug in the systemic circulation, accordingly promoting its targeted therapeutic effect^33^. Besides, the authors continued their study to investigate the proposed anti-SARS-CoV-2 activities of the aforementioned candidates by applying deep *in silico* studies towards the SARS-CoV-2 Mpro target receptor (6Y2G^24^) as a recommended mechanism of action. Collectively, the main pharmacophoric features of the co-crystallised inhibitor of SARS-CoV-2 Mpro could be suggested as follows (Figure 2):

- One functional group forms a hydrogen bond with GLU_166.
- A second functional group forms a hydrogen bond with CYS_145.
- An additional moiety to interact with HIS_41, HIS_163, HIS_164, or GLY_143.

The *N*-(5-nitrothiazol-2-yl) carboxamide scaffold was kept unchanged in all examined compounds. The authors mainly focussed on studying the influence of altering the size, type, and flexibility of the α-substituent to the carboxamide in addition to compound contraction on SARS-CoV-2 activity. In other words, the α-substituent group to the carboxamide (R) was designed in variable sizes hoping to fit properly inside the target receptor pocket which could improve the binding pattern accordingly.

## Materials and methods

### Materials

Egg yolk l-α-phosphatidylcholine (PC) (lecithin) and tristearin were purchased from Sigma-Aldrich, Inc. (St. Louis, MO). The Brij52 (polyoxyethylene (2) cetyl ether) was obtained from BASF Co. (Florham Park, NJ). The sodium chloride, potassium dihydrogen orthophosphate, methanol, sodium hydroxide, magnesium chloride, chloroform, and absolute ethanol were purchased from El-Gomhouria Chemical Co. (Cairo, Egypt). The dialysis membranes (Spectra/Pore^®^, cut-off 12 000–14 000) were purchased from Spectrum Laboratories Inc., Rancho Dominguez, CA). All chemicals and solvents were of analytical grade and were used as received.

### Methods

#### Fabrication of 3b-loaded emulsomes

**3b**-loaded EMLs were fabricated adopting the thin-film hydration technique with minor modification^36^. Briefly, in a round-bottom flask, 20 mg drug, 50 mg or 100 mg tristearin as lipid core, 25 mg or 50 mg PC, 20.0 or 40.0 mg Brij52, and 10.0 mg cholesterol were dissolved in 10 mL solvent mixture chloroform:methanol (2:1) (Table 1). Then, in a rotatory evaporator, the organic solvent was completely dispelled under reduced pressure at 60 °C for 30 min. The acquired dry film was then hydrated using 10 mL phosphate-buffered saline (PBS) at 60 °C (that exceeded the transition temperature of the lipid phase (Tc)) for 45 min. Finally, an ultrasonicator (Elmasonic, model LC 60/H, Wetzlar, Germany) was employed for further reduction in the particle size (PS) of the resulting EMLs dispersions kept at room temperature for 10 min.

**Table 1.** 2^3^ full factorial experimental designs; experimental runs, independent variables, and estimated responses of three loaded EMLs.

Formula	A Lipid core amount (mg)	B PC amount (mg)	C Brij 52 amount (mg)	Y1 (EE%)	Y2 (PS, nm)	Y3 (ZP, mV)	PDI
F1	50	25	20	65.4 ± 2.1	258.4 ± 13.8	–12.2 ± 1.4	0.34 ± 0.08
F2	100	25	20	83.5 ± 4.3	369.2 ± 21.9	–18.2 ± 2.9	0.45 ± 0.03
F3	50	50	20	76.8 ± 3.2	335.7 ± 27.1	–25.5 ± 2.2	0.37 ± 0.07
F4	100	50	20	93.3 ± 3.1	428.6 ± 24	–31.9 ± 4.7	0.61 ± 0.1
F5	50	25	40	54.7 ± 1.8	212.9 ± 17	–34.3 ± 1.9	0.24 ± 0.06
F6	100	25	40	78.9 ± 2.2	327.8 ± 10.4	–43.6 ± 3.4	0.31 ± 0.06
F7	50	50	40	63.1 ± 2.4	287.1 ± 19.8	–37.8 ± 4.2	0.52 ± 0.07
F8	100	50	40	86.5 ± 3.9	387.3 ± 15.4	–49.7 ± 5.8	0.23 ± 0.05

#### In vitro analysis and optimisation of 3b-loaded EMLs

##### Estimation of the entrapment efficiency percentage (EE%)

The percentage of compound **3b** enclosed within the fabricated EMLs was assessed in triplicates, where 1 mL aliquots of the EMLs dispersion were first diluted with 5 mL distilled water then agitated for 2 min, and finally, 1 mL of the final dispersion was then subjected to 1 h cooling centrifugation (Beckman Instruments, Fullerton, CA) at 15 000 rpm and kept at 4 °C^37^. The sedimented pellets were washed off twice with distilled water and recentrifuged for 30 min. The vesicles were disrupted using methanol and then sonicated for 2 h at room temperature. The analysis of the total concentration of embedded **3b** within the vesicles was conducted using UV spectrophotometry at *λ*max 254 nm versus methanol as the blanks. The EE% of the formulae was computed using the formula: EE% of **3b** entrapped = (amount of **3b** enclosed/overall amount of **3b**) × 100.

##### Estimation of zeta potential (ZP), PS, and polydispersity index (PDI)

The prepared **3b**-loaded EMLs were analysed in triplicates for determination of particle (or vesicle) size (PS), ZP, and PDI using a Zetasizer ZS (Malvern Instruments, Malvern, UK) (Table 1). 0.1 mL of the formulae dispersions were diluted to 10 mL with distilled water and vortexed for 5 min. The analysis was conducted at 25 °C using dynamic laser scattering with a 45 mm focus lens and a beam length of 2.4 mm^38^.

#### Experimental design and choice of the optimal 3b-loaded EMLs

Design Expert^®^ Version 13 (Stat Ease, Inc., Minneapolis, MN) was involved in the investigation of the influence of altering various formulation aspects of EMLs on their responses. Eight experimental runs were attained from the manipulated design adopting 2^3^ experimental designs. Three factors were considered as the independent variables: lipid core amount (A), PC amount (B), and Brij52 amount (C), where EE% (Y1), PS (Y2), and ZP (Y3) were set as the dependent variables. The selection of the optimal **3b**-loaded EML formulation was conducted based on the highest EE%, ZP, and lowest PS values. The consideration of the main effects and their relative significance were explored according to ANOVA statistical analyses. Furthermore, the optimal formulation with the highest desirability value was picked and involved in further assessments. Finally, the composition of the optimised formula was used in the preparation of the other compounds loaded emulsomal nanoparticles (EMLs) in order to investigate the effect of formulation of all compounds (**3a**–**g**) on their anti-SARS-CoV-2 activity which represent one of the prime targets of the study.

#### In vitro investigation of the optimum 3b-loaded EMLs

##### Lyophilisation of the optimised EML formula

Freeze-dryer (Alpha 2-4, CHRIST, Osterode am Harz, Germany) was employed in the solidification of the optimised **3b**-loaded EML formula. To impede the lysis and fusion of the vesicles, mannitol (5% w/v) was added as the cryoprotectant. The EML dispersion was kept overnight at −80 °C, and it was dried for 24 h under a vacuum. The attained emulsomal powder was preserved in a desiccator in tightly closed amber-coloured glass tubes for further characterisation.

##### Differential scanning calorimetry (DSC)

DSC (DSC-50, Shimadzu, Kyoto, Japan) was involved in the assessment of the thermal behaviour of each pure compound **3b**, plain optimum formula, and **3b**-loaded EMLs. The calibration of the instrument was conducted using purified indium (99.9%). The thermal behaviour of the samples was investigated in a temperature range of 20–400 °C at a scan rate of 10 °C/min under nitrogen^39^.

##### In vitro compound 3b release experiment

The release pattern of compound **3b** from the optimum formula compared to the drug dispersion was investigated as follows: 1 mL of the **3b**-loaded EML dispersions were diluted with 1 mL of Sorensen phosphate buffer (pH 7.4), then 1 mL of the produced dispersion (comprising 1 mg of **3b**) was added to a 2.5 cm diameter 10 cm glass cylinder closed from one side with a pre-soaked cellulose membrane. Then the glass cylinder was attached to the shaft of the dissolution tester (Copley, DIS 8000, Nottingham, UK) and left to rotate at a speed of 50 rpm in 900 mL of the same Sorensen phosphate buffer kept at 37 ± 0.5 °C. At different scheduled time intervals, a 5 mL sample was withdrawn and replaced with an equal volume of freshly prepared dissolution medium for keeping a constant volume and maintaining constant sink conditions. The percentages of **3b** released were measured spectrophotometrically at 254 nm and were computed in triplicate.

#### In vitro studies

##### MTT assay

To determine the cytotoxic concentration CC_50_ of each examined compound in VERO-E6 cells, the 3-(4,5-dimethylthiazol-2-yl)-2,5-diphenyltetrazolium bromide (MTT) assay was conducted as previously described^40^. The MTT assay method was performed with minor changes to calculate the newly synthesised candidates’ minimum concentrations that cause 50% toxicity to the cells (CC_50_). The full methodology was elucidated in the supplementary material (S1).

##### Inhibitory concentration 50 (IC_50_)

The IC_50_ for each examined compound formula (**F3a**–**g**), which is equivalent to the minimum concentration to inhibit the virus infectivity by 50% compared to the virus control, was calculated^41^. The complete methodology was depicted in the supplementary material (S2).

##### Mode of action against SARS-CoV-2

The possible mode of action for the most potent EMLs formula (**F3e**) towards SARS-CoV-2 inhibition was examined at three different stages of the virus propagation cycle and based on three main possible modes of action:

- The direct effect of each extract is to inactivate the virus viability (virucidal activity).
- The ability of each extract to inhibit the attachment of the virus to infected cells – membrane fusion is known to block the viral entry (viral adsorption).
- Inhibition of budding and viral replication.

Additionally, the above-mentioned modes of action could account for the recorded antiviral activities either independently or in combination. In this regard, the interaction between **F3e** and SARS-CoV-2 could be explained through the aforementioned three different modes of action. Therefore, the plaque infectivity reduction assay was performed according to the reported procedure (**S3**).

#### In silico studies

##### Docking studies

Molecular docking for the examined candidates (**3a**–**3g**) towards the binding site of SARS-CoV-2 Mpro was carried out using the MOE 2019.0102^42^. This was done to investigate the proposed SARS-CoV-2 Mpro inhibitory activity of the studied compounds compared to the co-crystallised (**Co**) inhibitor of the target receptor.

The aforementioned compounds were sketched individually in the working window of MOE, hydrogenated in 3D orientation, and energy minimised according to the described methodology^43^. All the prepared derivatives together with the **Co** inhibitor were inserted in one database to be ready for the docking process. Then, the X-ray structure of SARS-CoV-2 Mpro (PDB ID: 6Y2G^24^) was downloaded from the Protein Data Bank (www.rcsb.org). It was opened within the working window of MOE, corrected for the missed parts, and finally, energy was minimised as discussed earlier^44^. A general docking process was performed by inserting the previously mentioned database in place of the ligand icon and adjusting the program specifications as mentioned before^45^.

##### Molecular dynamics simulations and MM-GBSA calculations

The MD simulations^46^ were carried out through the Desmond package (Schrödinger LLC, New York, NY)^47^. Also, the MM-GBSA calculations of Schrodinger^48^ were applied to calculate the energies for all the studied complexes^49^. The full methodology is described in detail in the supplementary material (S4 and S5).

## Results and discussion

### Chemistry

Previously, **3a**–**g** were designed and synthesised by reacting *N*-acyl benzotriazoles (1**a**–**g**) and 5-nitrothiazol-2-amine (**2**) in the presence of TEA in acetonitrile at R.T. for 1 h as depicted in Scheme 1^31^.

### Experimental design and statistical evaluation

The investigation of the impact of the fabrication variables on the supposed responses was conducted using a 2^3^-full-factorial design. The composition of the eight attained experimental runs and their related EE, PS, and ZP responses are demonstrated in Table 1. The suitability of the model to manoeuvre the design space was deduced using model precision value^36^. As shown in Table 2, a precision value exceeding four was perceived for all the dependent variables. Furthermore, the gap between predicted and adjusted *R*^2^ should not exceed a value of 0.20 to confirm a reasonable agreement. From Table 2, it can be depicted that the predicted *R*^2^ values were consistent with adjusted *R*^2^ values for all the dependent variables. Based on the superiority of previously reported IC_50_ against SARS-CoV-2, compound **3b** was selected as a drug model to be endorsed in the formulation and optimisation of fabrication variables^31^.

**Table 2.** 2^3^ Output factorial analysis data of **3b**-loaded EMLs and the predicted, observed responses, and deviation percent of the Optimum formula (F6).

Responses	EE (%)	PS (nm)	ZP (mV)
*R* ^2^	0.9999	1	0.9995
Adjusted *R*^2^	0.9995	0.9997	0.9962
Predicted *R*^2^	0.9957	0.9977	0.9965
Adequate precision	146.6	204.5	50.7
Significant factors	X1, X2	X1, X2	X1, X2
Observed value of the optimal formula (F6)	78.9	327.8	–43.6
Predicted value of the optimal formula (F6)	79	328.2	–43.87
Absolute deviation %	0.126	0.122	0.62

### Influence of the compounding variables on EE%

**Figure 3.**
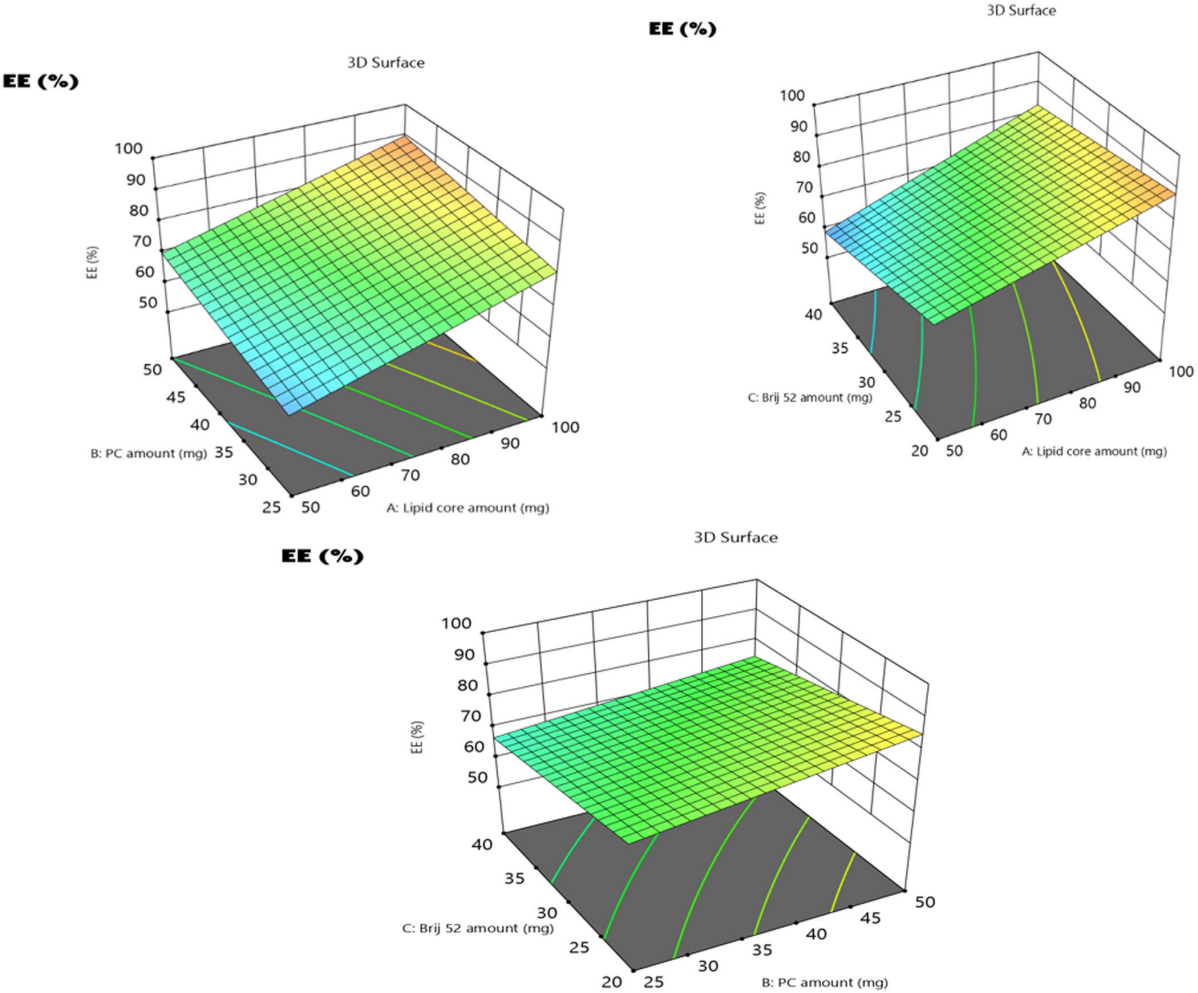
3D surface plot of the impact of (A) lipid core amount, (B) PC amount, and (C) Brij 52 amount on EE% of **3b**-loaded EMLs.

The assessment of the EE% is beneficial in the estimation of the loading capability of compound **3b** within the formulated vesicles. The encapsulation percentages of **3b** within the vesicles ranged from 63.1 ± 2.4 to 93.3 ± 3.1%. In addition, the model with all variables for lipid core amount (factor A), PC amount (factor B), and Brij52 amount (factor C) significantly impacted (*p* = 0.0153) the EE%, as illustrated in the 3D plots (Figure 3).

The increase in the amount of the lipid core (factor A) from 50 mg to 100 mg resulted in a significant (*p* = 0.0062) improvement in the entrapment of compound **3b**. It was previously stated that the increase in the lipid core amount leads to an increase in the lipophilic space within the vesicles which will accommodate the drug^50^. Furthermore, the viscosity of the fabricated vesicular dispersion was elevated with the increase in the amount of the lipid core; thus, the boosted viscosity can hinder the drug diffusion to the external hydrophilic phase, leading to a higher EE%^51^. Moreover, increasing the amount of PC (factor C) from 25 to 50 mg predisposed to a significant increase (*p* = 0.0137) in the EE%. The increase in PC content resulted in the assembly of PC multilayers around the lipid core, thus allowing the drug to be efficiently enclosed within these bilayers. In addition, a higher amount of PCs led to the subsequent enhancement in the vesicle rigidity and compactness, thus impeding drug leakage^52^. ANOVA statistical analysis declared a significant negative impact on EE% (*p* = 0.0142) upon increasing the amount of Brij from 20 mg to 40 mg. This could be credited to the development of more pores (voids) in the bilayers; moreover, the elevation in the amount of Brij predisposed to an increase in the vesicular fluidity, thus resulting in increased drug leakage and subsequent diminished EE% values.

### The polydispersity index and the influence of the compounding variables on particle size

**Figure 4.**
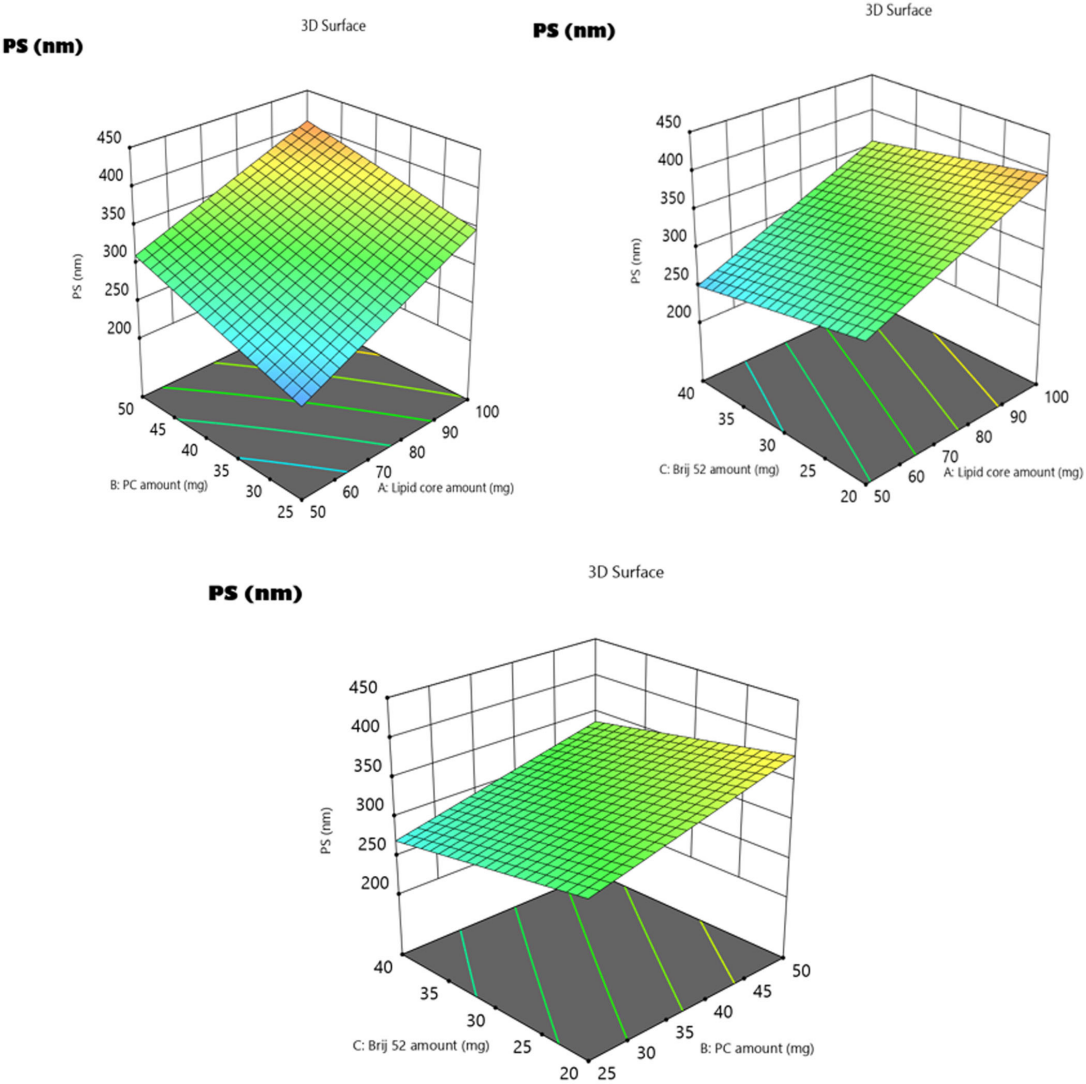
3D surface plot of the impact of (A) lipid core amount, (B) PC amount, and (C) Brij 52 amount on PS of **3b**-loaded EMLs.

The extent of sample homogeneity along with the level of monodispersity can be deduced via PDI values, whereas the PDI values getting close to zero indicate monodispersity, while those getting close to 1 indicate PDI. The PDI values of the **3b**-loaded EMLs shown in Table 1 ranged from 0.24 ± 0.06 to 0.61 ± 0.1. Thus, the values of the PDI for the prepared formulae shifted towards PDI, but within an appropriate range^53^. Generally, the drug’s permeation across intestinal membranes along with its fate and duration in blood circulation is strongly related to PS. Thus, promoted drug permeation, prolonged retention time, and boosted therapeutic activity are the significant results of decreasing the PS. As illustrated in Table 1, Y2, the PS of the fabricated **3b**-loaded EMLs ranged from 212.9 ± 17 to 428.6 ± 24 nm. The significance of the whole model with all three variables (factors A–C) was confirmed based on ANOVA analysis; this is graphically illustrated in the 3D plots (Figure 4). The rationale behind the effect of each variable on the PS can be discussed as follows.

Regarding the lipid core amount (factor A), the increase in the lipid core amount from 50 mg to 100 mg predisposed to a prominent increase in PS (*p* = 0.0049). This may be attributed to the supposed increase in the viscosity of the fabricated emulsion resulting from the elevation in the amount of lipid involved in the formulation^36^. Also increasing the amount of PC (factor B) resulted in a subsequent significant elevation in PS (*p* = 0.0075). This finding may be due to the increase in the thickness of the attained PC multiple bilayers which surround the lipidic core and this came in harmony with the results of Aldawsari et al., who studied the impact of the increase in the amount of the phospholipid on the PS of the fabricated raloxifene EMLs^33^. Moreover, the alignment of the PC at the interface between aqueous and oily phases provides the vesicles with the extra capability to endorse higher amounts of the target drug, leading to greater sizes of the formulated EMLs^52^.

On the other hand, the increase in the amount of Brij (factor C) led to a significant suppression in vesicle PS (*p* = 0.0115). Owing to the surface active properties of Brij, the higher amount of Brij will consequently diminish the interfacial tension of the system and alter the alignment t of the attained vesicles^54^. Additionally, the assembly of the Brij molecules at the surface of the vesicle sterically stabilises the vesicles by prohibiting their aggregation and improving their stability^36^.

### The influence of the compounding variables on zeta potentials

**Figure 5.**
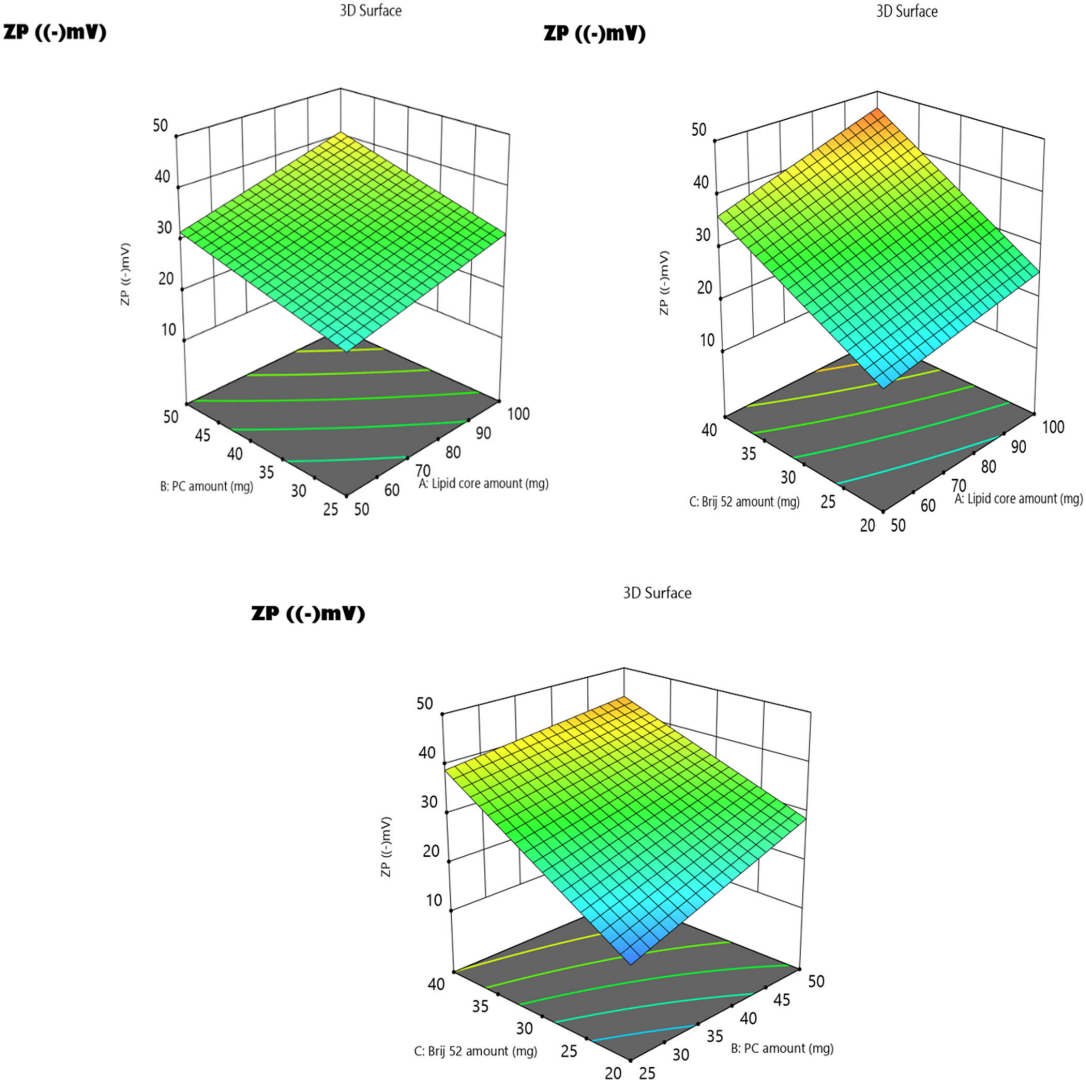
3D surface plot of the impact of (A) lipid core amount, (B) PC amount, and (C) Brij 52 amount on ZP of **3b**-loaded EMLs.

Based on the ZPs values, the stability of the vesicular system can be deduced, where ZP values fall in the range of ±30 mV and are assumed to attain adequate electrostatic stabilisation^55^. This guarantees a sufficient electric repulsion to be produced between the vesicles, thus impeding their agglomeration and promoting their stability^56^. In the present study, ZP values of the prepared EMLs ranged from −12.2 ± 1.4 to −49.7 ± 5.8 mV (Table 1). It was found that ZP was significantly influenced by all the variables (factors A–C) as depicted from the results of ANOVA and displayed as 3D plots (Figure 5). The influence of each variable can be justified as follows.

According to ANOVA results, the increase in lipid core amount (factor A) led to a significant increase in ZP as an absolute value (*p* = 0.041). Increasing the amount of lipid permitted the diffusion of a larger amount of negatively charged drug into the lipid core, thus imparting higher negative charges imparted on the surface of the EMLs. Furthermore, increasing the amount of PC (factor B) from 25 to 50 mg predisposed to a significant increase (*p* = 0.038) in ZP values as an absolute value. The same findings were previously reported by El Zaafarany et al., who correlated this increase to the arrangement of the negatively charged PC on the outer surface^52^. Consequently, the elevation in the amounts of PC increased the overall values of the ZPs^33^. Additionally, ANOVA results revealed that the elevation in the amount of Brij52 (factor C) prominently increased (*p* = 0.018) the overall ZP values. This may be attributed to the alignment of a higher number of negatively charged PEG moieties as an electronegative coat that wraps the vesicles on increasing the amount of Brij 52; hence, elevation of the overall ZP values was noticed^57^.

### Selection and validation of the optimal 3b-loaded EMLs formulation

The selection of the optimal formula along with the investigation of the impact of the formulation variables on the characteristics of the fabricated vesicles was carried out based on the statistical analysis utilising Design Expert^®^. The formula (F6) composed of 100 mg of the lipid core, 25 mg of PC, and 40 mg of Brij52 was found to be the optimal formulation as it has the highest desirability value (0.628). The composition of the optimal formula was then used in the formulation of the rest compounds’ EMLs. Additionally, assessing the percentage differences between the observed and predicted values of the selected responses; EE%, PS, and ZP were involved in assuring the validities of the models tested. As noticed in Table 2, the low deviation percentage as an absolute value (less than 10%) between the predicted and observed values for all responses emphasises the suitability of the statistical design in the analysis of the findings^36^.

### In vitro investigations of the optimum 3b-loaded EML formula

#### Differential scanning calorimetry

**Figure 6.**
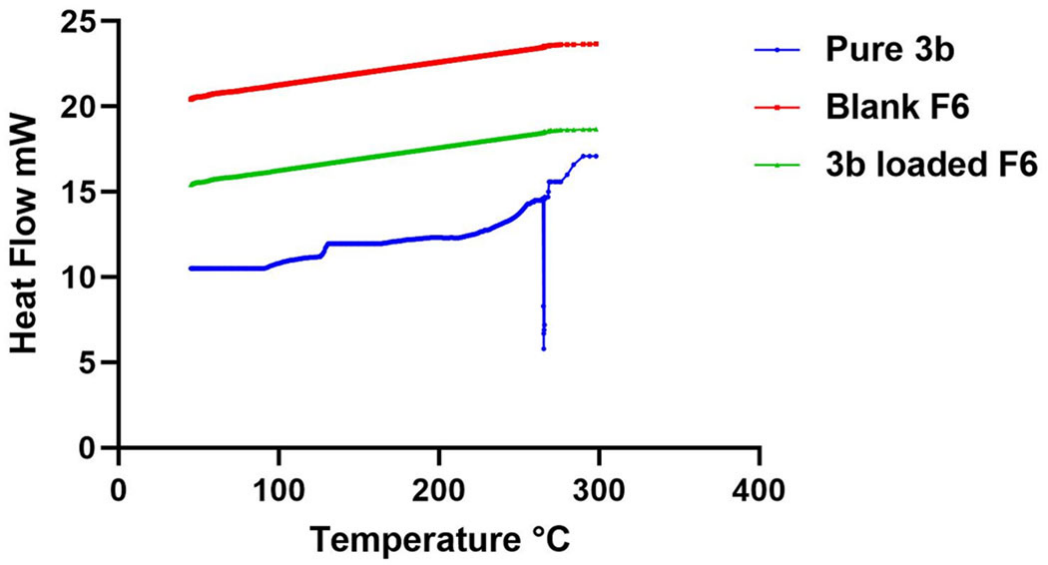
DSC thermograms of pure compound **3b**, blank F6, and **3b**-loaded F6.

DSC study was conducted in order to estimate the change in the physical state and the degree of crystallinity of pure compound **3b** compared to **3b**-loaded EMLs. Figure 6 displays the thermal behaviour of the pure **3b** compared to that of the blank lyophilised formula and optimum lyophilised **3b**-loaded optimum EMLs (F6). Compound **3b** discloses a sharp characteristic endothermic peak at 265.2 °C which is related to compound **3b**’s melting point. Meanwhile, the thermograms of both lyophilised blank formula and F6 disclosed the disappearance of any characteristic peak of either compound **3b** or the components incorporated in the vesicular formulation. Accordingly, this affirmed the total transformation of the drug along with the other components of the formula to an amorphous state instead of the crystalline one, implying the capability of the formulated vesicles to enclose the drug.

#### Comparative 3b in vitro release experiment

**Figure 7.**
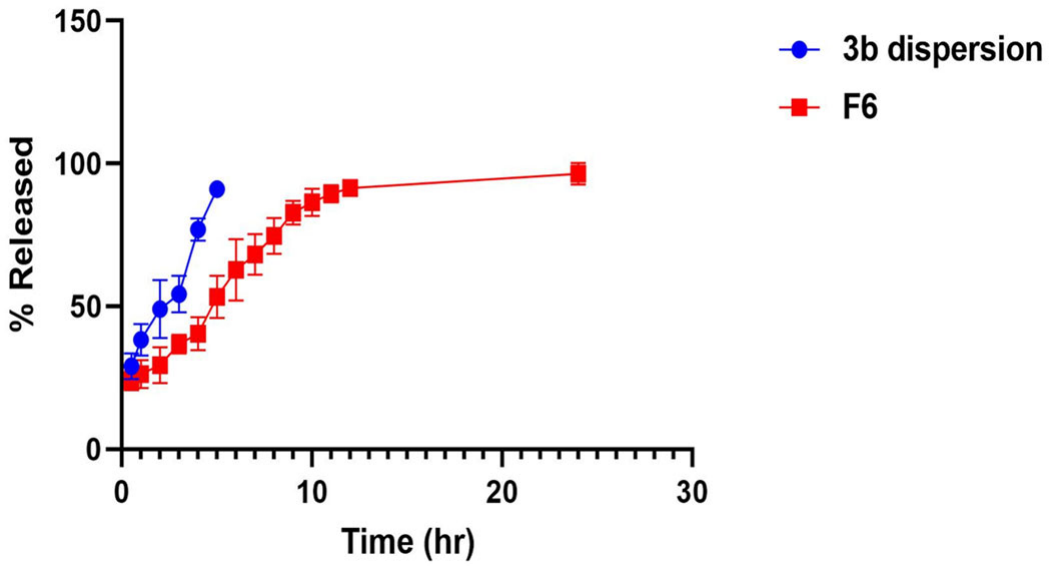
*In vitro* drug release profile of **3b** from optimised formula F6 compared to the drug dispersion.

Figure 7 reveals a comparative release profile of the optimised **3b**-loaded EML formula (F6) relative to **3b** dispersion to denote the release pattern of the drug and the degree of stability of the prepared vesicles. The release pattern of the drug from F6 exhibited more controlled and prolonged release relative to the drug dispersion over 24 h, where the cumulative amount released from the optimised formula F6 after 24 h was 96.2% ± 5.1%. This can be rationalised by the drug pool effect of the vesicles enclosing the drug from which the drug was released in two successive phases: the first rapid burst release followed by the delayed and more controlled phase^58^. Additionally, the PEGylated coat resulted from Brij 52 inclusion in the formulation, which wrapped the bilayer and imparted more drug solubilisation and a higher release rate owing to the solubilisation effect and elevated hydrophilicity of PEG moieties^59^. Meanwhile, in the case of **3b** dispersion, the drug was mostly released after 5 h.

### In vitro studies

#### SARS-CoV-2 inhibitory assay

**Figure 8.**
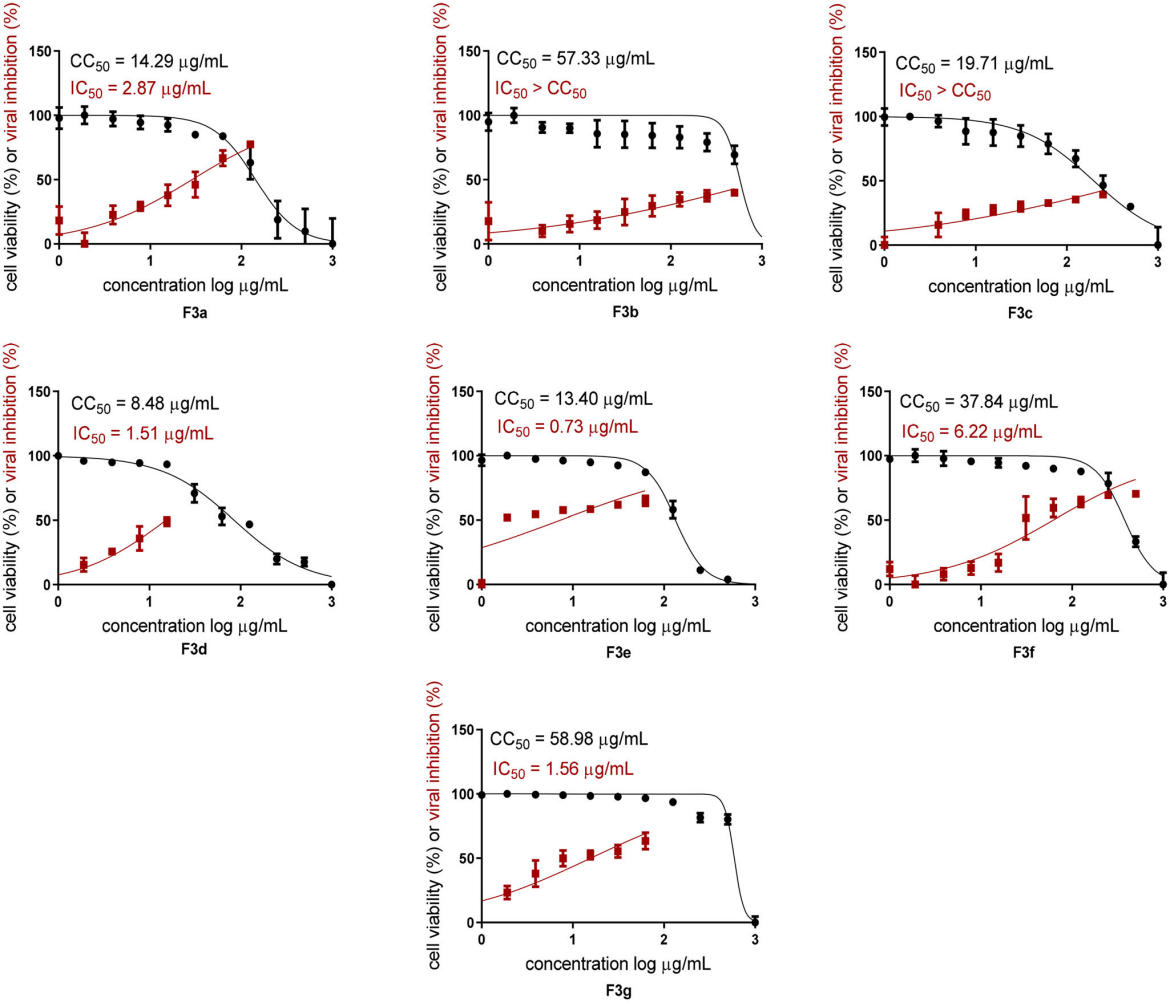
*In vitro* assessment of cytotoxicity and anti-SARS-CoV-2 activity of **F3a**–**g** in Vero E6 cells via MTT assay (*h*CoV-19/Egypt/NRC-03/2020 (accession number on GSAID: EPI_ISL_430820)).

Both cytotoxicity and anti-SARS-CoV-2 activity of all examined compounds loaded EMLs (**F3a**–**g**) were assessed in Vero E6 cells via MTT assay. Half maximal cytotoxic concentrations (CC_50_) were 14.29 (**F3a**), 57.33 (**F3b**), 19.71 (**F3c**), 8.48 (**F3d**), 13.4 (**F3e**), 37.84 (**F3f**), and 58.98 μg/mL (**F3g**). In addition, half maximal inhibitory concentrations (IC_50_) were calculated using dose–response curves as follows; 2.87 (**F3a**), 1.51 (**F3d**), 0.73 (**F3e**), 6.22 (**F3f**), and 1.56 μg/mL (**F3g**). On the other hand, both **F3b** and **F3c** showed higher IC_50_ values compared with their respective CC_50_ values, Figure 8 and Table S1 (supplementary material). The most potent **F3e** elicited a selectivity index (SI = CC_50_/IC_50_) of 18 while **F3g** demonstrated the highest SI reaching 37.

On the other hand, both **F3b** and **F3c** showed higher IC_50_ values compared with their respective CC_50_ values undermining their potential as anti-SARS-CoV-2 therapeutic agents.

The enhanced antiviral activity of the compounds after formulation may be attributed to the capability of attachment of the tailored vesicles to viral cells either by endocytosis or by fusion. This conjugation aided in the accumulation and creation of concentration and thermodynamic gradient at the viral membrane, accordingly, predisposed to boosted penetration of the compounds across virus cellular membranes. Moreover, the lipids and surfactant incorporated in the formulation of EMLs conveyed a vital in enhancing the compound’s antiviral activity, as they augment the diffusion of the drugs through the viral cells^36^.

#### Mode of action against SARS-CoV-2

**Figure 9.**
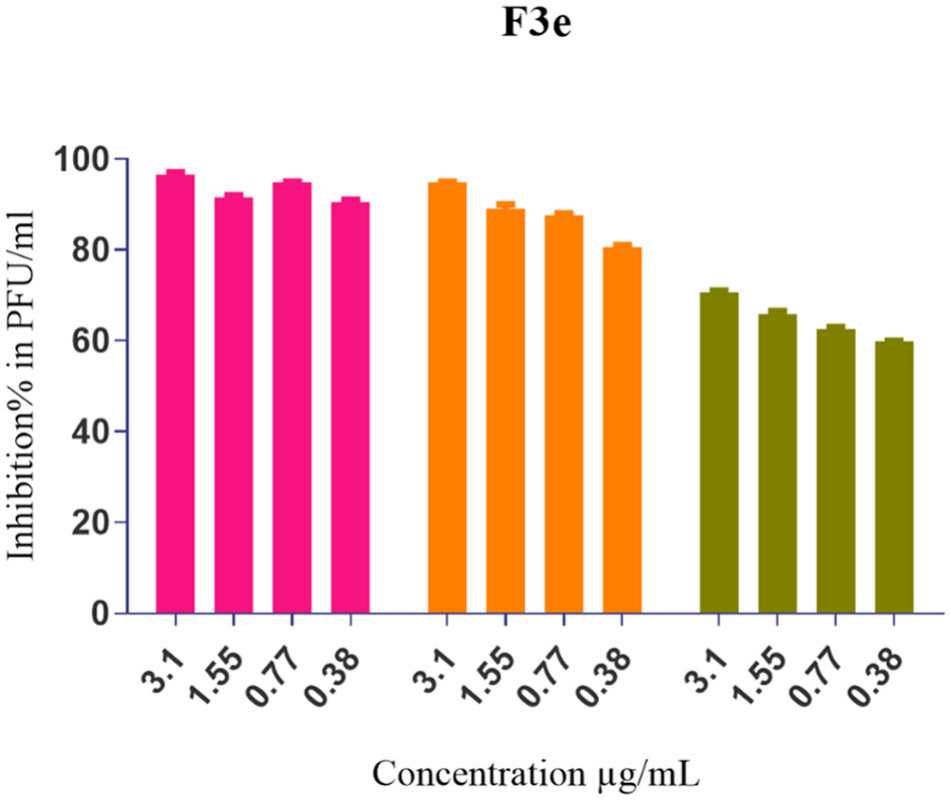
Mode of action of the most potent formulation (**F3e**) against SARS-CoV-2.

The most potent hit against SARS-CoV-2 (**F3e**) was selected for further elucidation of a possible mode of action. Using an array of four different safe concentrations, three potential anti-viral mechanisms were investigated; direct virucidal activity, blockade of viral adsorption to host cell receptors, and inhibition of intracellular viral replication. Results showed that **F3e** exhibited a combination of virucidal (>90%), viral adsorption (>80%), and viral replication (>60%) inhibition as the principal mechanisms against SARS-CoV-2 (Figure 9).

### In silico studies

#### Molecular docking studies

A molecular docking study for the studied compounds (**3a**–**3g**) towards the SARS-CoV-2 Mpro target (6Y2G^24^) was performed to investigate their expected inhibitory effects. Besides, the co-crystallised inhibitor (**Co**) of the target receptor was inserted as a reference standard. Briefly, the most active candidates (**3d**, **3e**, and **3g**) and **Co** were selected for further investigations.

The docked **Co** of SARS-CoV-2 Mpro formed two hydrogen bonds with the crucial amino acids (GLU_166 and GLN_192) important for producing the antagonistic effect within the binding pocket of SARS-CoV-2 Mpro. Its binding score was recorded to be −8.19 kcal/mol (RMSD = 1.66 Å). On the other hand, compound **3d** was able to form two hydrogen bonds with the same crucial amino acid (GLU_166), and its binding score was found to be −7.32 kcal/mol (RMSD = 1.75 Å). Moreover, compound **3e** was observed to form one hydrogen bond with the first crucial amino acid (GLU_166). Also, it formed two pi-hydrogen interactions; one of them with the second crucial amino acid (GLN_192) and the other one with ALA_191 amino acid. **3e** achieved a binding score of −7.50 kcal/mol (RMSD = 1.24 Å). Furthermore, compound **3g** formed two hydrogen bonds with the same crucial amino acid (GLU_166), and also it bound another crucial amino acid (HIS_41) with a pi–pi bond. The binding score of **3g** was observed to be −7.79 kcal/mol (RMSD = 1.77 Å) (Table 3).

**Table 3.** 3D interactions and positioning of the **Co** inhibitor of SARS-CoV-2 Mpro (PDB ID: 6Y2G) binding pocket and the most active candidates (**3d**, **3e**, and **3g**).

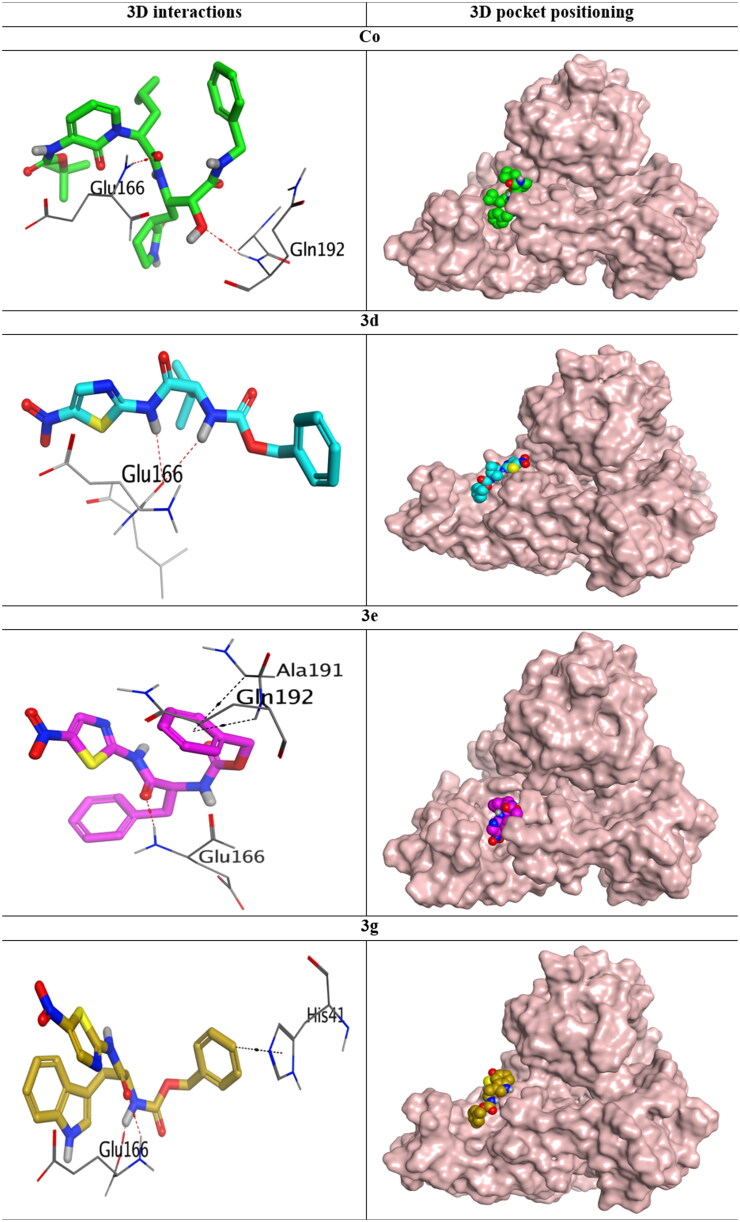	

#### Molecular dynamics simulations

To investigate the exact behaviour of the examined candidates towards the binding pocket of SARS-CoV-2 Mpro, MD simulations were performed. MD studies were applied for 200 ns and under the same conditions of the physiological environment. All the tested docked complexes were subjected to MD simulations for 200 ns and compared to the co-crystallised ligand (**Co**).

##### RMSD analysis

The RMSD was described to compare the deviation degree of the studied protein complexes compared to its initial one in a quantitative manner. This is important to judge each system’s stability during the simulation time.

**Figure 10.**
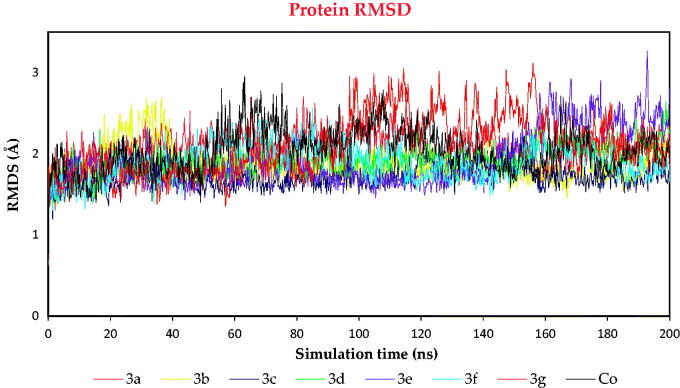
The RMSD of complexes (**3a**, **3b**, **3c**, **3d**, **3e**, **3f**, **3g**, and **Co**) for SARS-CoV-2 Mpro as a function of simulation time (200 ns).

All the studied eight protein complexes showed promising RMSD values <2.7 Å, except for **3e** and **3d** protein complexes which showed RMSD values <3.2 Å. This indicates good stability behaviours for all the examined complexes during the 200 ns of the simulation time (Figure 10).

**Figure 11.**
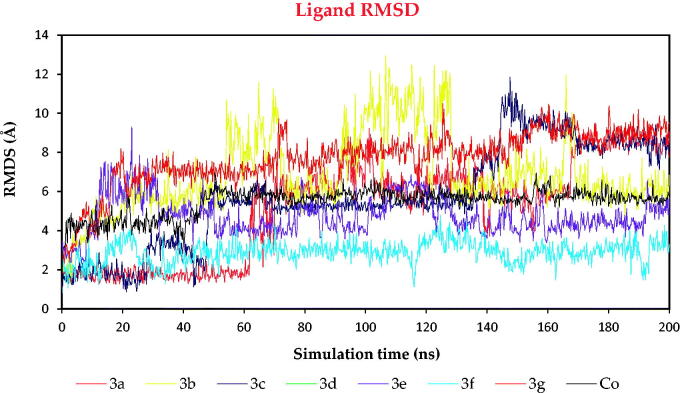
The RMSD of ligands (**3a**, **3b**, **3c**, **3d**, **3e**, **3f**, **3g**, and **Co**) for SARS-CoV-2 Mpro, respectively, as a function of simulation time (200 ns).

On the other hand, to compare the exact behaviour of each ligand within the binding pocket of SARS-CoV-2 Mpro, the ligand RMSD values within protein complexes were recorded as well (Figure 11). It was clear that ligands **3a**, **3b**, **3c**, **3d**, **3e**, **3f**, **3g**, and **Co** moved inside the binding pocket of Mpro within the ranges of 9, 12.5, 11, 6.4, 9, 4.8, 10.5, and 7.2, respectively.

The RMSD behaviours of **3d**, **3e**, and **3g** were selected due to their superior biological activities – to be compared to that of **Co** and discussed in more detail.

Ligand **3d** showed very stable behaviour within the binding pocket of SARS-CoV-2 Mpro from the start till the end of the simulation time. It fluctuated within the range of 3.2 Å, indicating superior stability compared to that of the docked **Co**. Where the RMSD of **Co** showed lower stability and fluctuated within the range of 4 Å during the 200 ns of the simulation time. However, ligand **3e** showed good stability and fluctuated within the range of 6 Å. It showed the highest fluctuations from 10 to 35 ns of the simulation time, where it returned to stable behaviour till the end of the simulation time. Besides, ligand **3g** showed moderate stability where it fluctuated within the range of 7 Å, where it showed higher values from 100 to 200 ns of the simulation time (Figure 11). Based on the above discussion, we can conclude the very promising behaviour of the aforementioned ligands (**3d**, **3e**, and **3g**) with respect to that of **Co**.

Figure S1 (supplementary material) reports the RMSF (root mean square fluctuation), where most proteins fluctuated within the range of 4–5 Å, with *C*- and *N*-terminals fluctuating at around 7 Å. Such change is acceptable within such a large system.

##### Binding interactions analysis (histogram and heat map)

**Figure 12.**
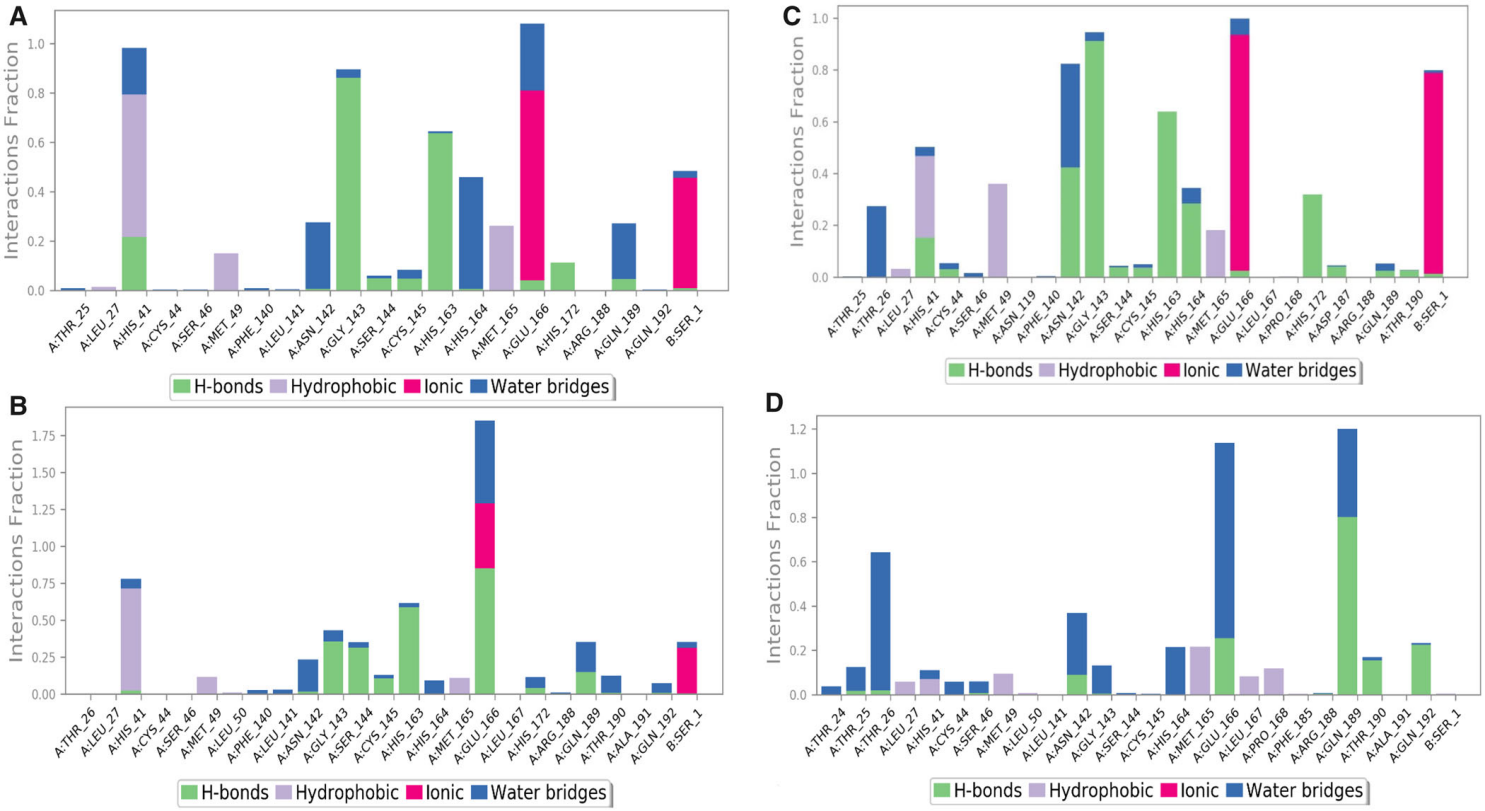
Histogram describing the binding interactions between the SARS-CoV-2 Mpro and its ligand during the simulation time of 200 ns for (**A**) **3d**, (**B**) **3e**, (**C**) **3g**, and (**D**) **Co**.

First, the histograms of the three selected complexes (**3d**, **3e**, and **3g**) besides that of **Co** were analysed to describe the SARS-CoV-2 Mpro-ligand interactions during the simulation time of 200 ns (Figure 12).

**3d**-complex histogram showed that GLU_166, HIS_41, and GLY_143 contributed with 110, 100, and 90% of the interactions. GLU_166, HIS_41, and GLY_143 contributed mainly through ionic bonds (>70%), hydrophobic interactions (60%), and hydrogen bonds (>80%), respectively (Figure 12(A)). Besides, the **3e**-complex histogram represented that GLU_166 was the main amino acid that contributed to the interactions (180%) through hydrogen bonds (80%), ionic bonds (45%), and water bridges (55%) followed by HIS_41, which contributed by 75% through hydrophobic interactions mainly (Figure 12(B)). Moreover, the histogram of **3g**-complex showed that GLU_166 was the principal amino acid that contributed to the interactions (100%) through hydrogen bonds (<5%), ionic bonds (>90%), and water bridges (5%). Also, GLY_143 contributed with >90% of the interactions mainly through hydrogen bonds (Figure 12(C)). On the other hand, the **Co**-complex histogram showed that GLN_189 was the main amino acid in the interactions (120%) through hydrogen bonds (80%) and water bridges (40%). However, GLU_166 was the second amino acid that contributed to the interactions (110%) through hydrogen bonds (20%) and water bridges (90%), as depicted in Figure 12(D). According to the previous discussion, we can conclude the great significance of GLU_166, HIS_41, and GLY_143 (especially GLU_166) in the binding pocket of SARS-CoV-2 Mpro. Therefore, the superior anti-SARS-CoV-2 biological effects of the studied compounds towards the binding site of SARS-CoV-2 Mpro can be justified.

**Figure 13.**
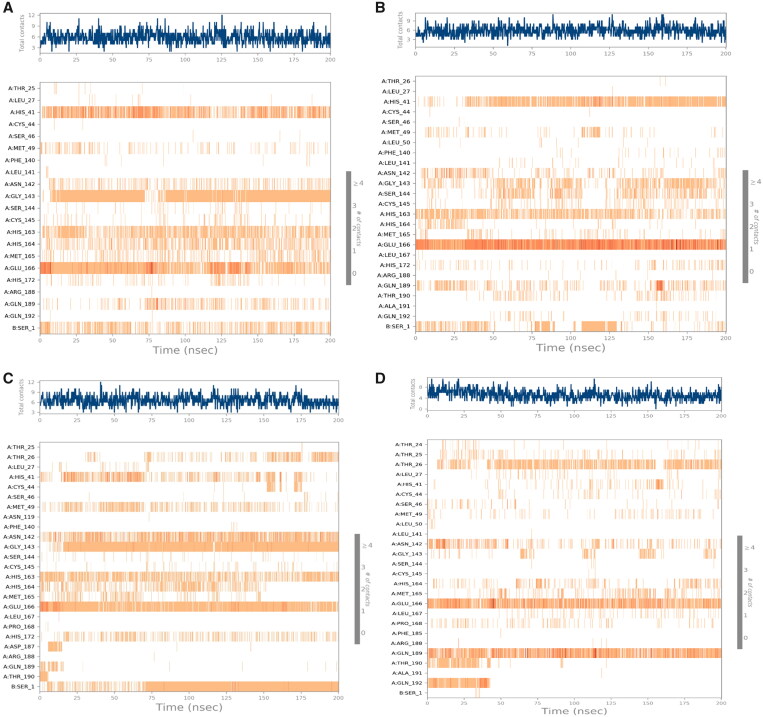
Heat map showing the total number of SARS-CoV-2 Mpro-ligand interactions all over the simulation time of 200 ns for (**A**) **3d**, (**B**) **3e**, (**C**) **3g**, and (**D**) **Co**.

On the other hand, the heat maps which describe the total contacts with respect to the simulation time (200 ns) for the four studied complexes (**3d**, **3e**, **3g**, and **Co**) are represented in Figure 13.

For the **3d**-complex, it was clear that GLU_166 and HIS_41 interactions were all over the simulation time. However, GLY_143 interactions were nearly absent before 10 ns and from 75 to 85 ns (Figure 13(A)**)**. Moreover, the heat map of the **3e**-complex showed that the binding interactions of GLU_166 extended throughout the whole time of the simulation. But, HIS_41 interactions started to be intense from 35 ns till the end of the simulation time (Figure 13(B)). Furthermore, the **3g**-complex heat map showed that GLU_166 interactions were intense from the beginning till the end of the simulation time. Besides, the binding interactions of GLY_143 started to be more intense from 20 ns till the end of the simulation time (Figure 13(C)). Finally, for the **Co**-complex histogram, it was obvious that both GLN_189 and GLU_166 contributed to the interactions from the beginning till the end of the simulation time (Figure 13(D)).

#### MM-GBSA calculations

The average MM-GBSA binding energies^60^ for all the studied complexes were calculated using the thermal_mmgbsa.py python script of Schrodinger^48^. Table 4 represents the Δ*G* binding, hydrogen-bonding, lipophilic, covalent binding, Coulomb, van der Waals, and the generalised Born electrostatic solvation energies for all complexes.

**Table 4.** Prime MM-GBSA energies for complexes (**3a**, **3b**, **3c**, **3d**, **3e**, **3f**, **3g**, and **Co**) of SARS-CoV-2 Mpro protein.

Complex	Δ*G* binding	Coulomb	Covalent	H-bond	Lipo	Bind packing	Solv_GB	VdW	St. Dev.
**3a**	–39.96	–3.19	0.72	–1.00	–9.75	–1.46	8.11	–33.39	6.39
**3b**	–52.27	–16.59	2.61	–1.67	–9.39	–1.50	16.47	–42.19	4.88
**3c**	–43.63	–7.62	1.47	–1.21	–8.18	–1.27	10.96	–37.77	7.71
**3d**	–56.97	–13.63	4.44	–1.61	–11.00	–1.54	15.00	–48.61	4.12
**3e**	–58.73	–0.89	1.10	–1.35	–14.52	–0.99	8.88	–50.94	4.81
**3f**	–53.05	–6.66	3.36	–1.54	–13.62	–2.26	12.95	–45.28	3.11
**3g**	–51.81	–15.55	4.60	–1.69	–11.82	–2.45	23.92	–48.81	4.96
**Co**	–55.25	–11.77	1.59	–0.63	–15.38	–1.48	23.25	–50.81	5.10

Coulomb: Coulomb energy; covalent: covalent binding energy; H-bond: hydrogen-bonding energy; Lipo: lipophilic energy; Solv_GB: generalised Born electrostatic solvation energy; VdW: van der Waals energy; St. Dev.: standard deviation.

Based on the introduced results in Table 4, we can observe that both compounds **3d** and **3e** achieved superior Δ*G* binding energies (–56.97 and −58.73 kcal/mol, respectively) compared to that of **Co** inhibitor (–55.25 kcal/mol). This indicates better stability for **3d** and **3e** within the binding pocket of SARS-CoV-2 Mpro compared to its **Co** inhibitor. Moreover, compounds **3b**, **3d**, and **3g** showed superior Coulomb energies (–16.59, −13.63, and −15.55 kcal/mol, respectively) compared to that of the **Co** inhibitor (–11.77 kcal/mol). The best covalent energies were recorded for **3d**, **3f**, and **3g** (4.44, 3.36, and 4.60 kcal/mol) compared to that of the **Co** inhibitor (1.59 kcal/mol). Notably, all compounds (**3b**–**3g**) showed better hydrogen-bonding energies with respect to that of the Co inhibitor. Besides, the bind packaging energies for compounds **3b**, **3d**, **3f**, and **3g** (–1.50, −1.54, −2.26, and −2.45 kcal/mol, respectively) exceeded that of the **Co** inhibitor (–1.48 kcal/mol) as well. However, only compound **3g** achieved the superior generalised Born electrostatic solvation energy (23.92 kcal/mol) compared to the **Co** inhibitor (23.25 kcal/mol). Finally, the best van der Waals energy was recorded for compound **3e** (–50.94 kcal/mol) compared to the **Co** inhibitor (–50.81 kcal/mol). Therefore, these findings confirm the superior affinities and subsequent intrinsic activities of the examined compounds (especially **3d**, **3e**, and **3g**) towards the binding pocket of SARS-CoV-2 Mpro.

## Structure–activity relationship (SAR) study of the examined compounds loaded EMLs (F3a–g)

**Figure 14.**
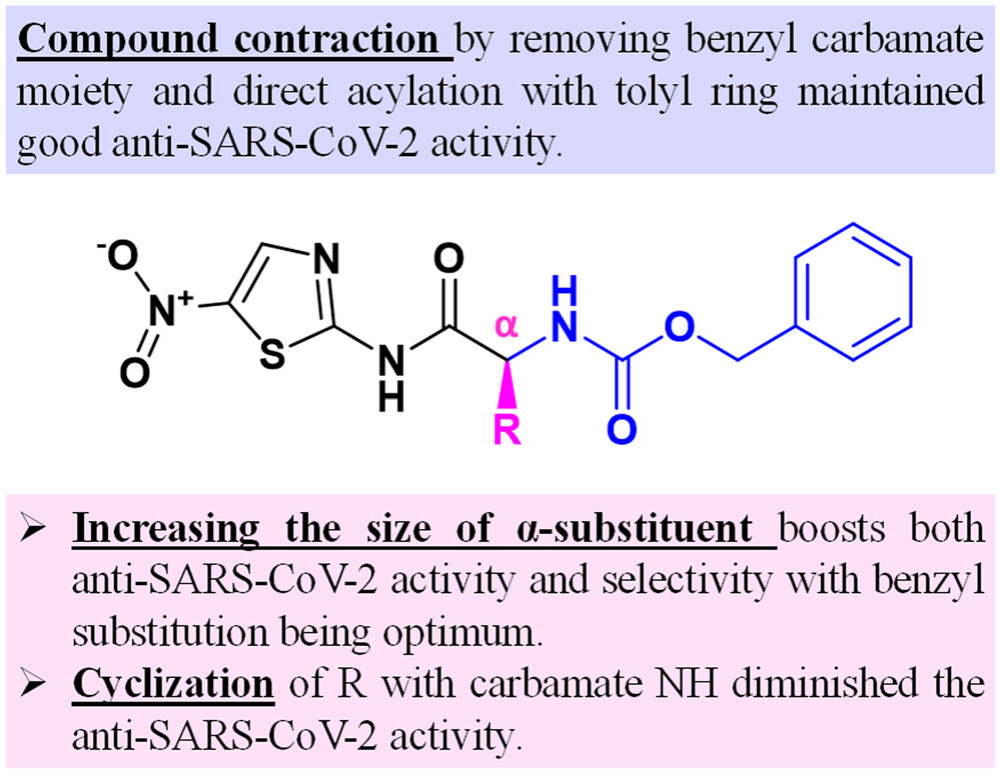
SAR summary for the synthesised *N*-(5-nitrothiazol-2-yl)-carboxamido derivatives as potential inhibitors of SARS-CoV-2 Mpro.

The *N*-(5-nitrothiazol-2-yl) carboxamide scaffold was kept unchanged in all examined compounds. We mainly focussed on studying the influence of altering the size, type, and flexibility of the α-substituent to the carboxamide in addition to compound contraction on SARS-CoV-2 activity. Based on both the *in vitro* (Figure 8) and the *in silico* results (Tables 3 and 4 and Figure 11), the following SAR conclusions can be made (summarised in Figure 14).

### Effect of α-substituent size

An increase in the size of substituent probed at the α-position to the *N*-(5-nitrothiazol-2-yl)-carboxamide scaffold was generally accompanied by a boost in the anti-SARS-CoV-2 activity. When compared to the unsubstituted compound **3b** (IC_50_ = 116.8 μg/mL), substitution with an aliphatic α-methyl group (compound **3c**) led to an almost twofold increase in SARS-CoV-2 inhibitory activity (IC_50_ = 63.13 μg/mL). Interestingly, a 77-fold drop in IC_50_ was observed upon substitution with the aliphatic but bulkier α-isopropyl group (compound **3d**) with an IC_50_ reaching 1.51 μg/mL. The use of bulkier aromatic α-substituents maintained such a boost in SARS-CoV-2 inhibitory activity where compound **3g** with methylene-(*1H*-indole-3-yl) group exhibited an IC_50_ of 1.56 μg/mL while employing a benzyl substituent in compound **3e** granted the best anti-SARS-CoV-2 activity among all examined candidates (IC_50_ = 0.73 μg/mL). Furthermore, enlarging the α-substituent size was found to induce better selectivity profiles for compounds **3d**, **3e**, and **3g** (selectivity indices; 5.61, 18, and 37, respectively). On the contrary, compounds **3b** and **3c** possessed higher IC_50_ values than their corresponding CC_50_ values, further thwarting their applicability as anti-SARS-CoV-2 agents.

### Effect of α-chain cyclisation

Chain cyclisation is one of the most commonly adopted drug design strategies as it may enhance binding affinity and/or stabilise target binding patterns^61^. Herein, we investigated the effect of cyclising the benzyl carbamate NH with the α-carbon via a propyl spacer forming the conformationally restricted pyrrolidine moiety (compound **3f**). Unfortunately, such a rigidification did not improve the anti-SARS-CoV-2 activity where **3f** demonstrated an IC_50_ of 6.22 μg/mL which is almost fourfold higher than compound **3d** having analogous side chain carbons. This could be attributed to a cyclisation-induced twist in the main scaffold that led to the deviation of compound **3f** away from key residues in the CYS-HIS dyad of the SARS-CoV-2 Mpro active site. Moreover, the capability to form an H-bond with the crucial amino acid GLU_166 was lost by cyclisation (supplementary material, Figure S2), further contributing to the observed diminished activity of compound **3f**.

### Effect of compound contraction

The effect of removing the benzyl carbamate moiety and directly acylating the aminothiazole with a tolyl ring was explored (compound **3a**) as well. Intriguingly, a good SARS-CoV-2 inhibitory potency was achieved with an IC_50_ of 2.87 μg/mL. One plausible justification for this is that the essential H-bond with CYS_145 was maintained (supplementary material, Figure S3). In addition, compound extension to reach border residues such as GLN_192, ALA_191, and HIS_41 is seemingly not crucial for anti-SARS-CoV-2 activity as the contraction in compound **3a** was quite tolerated.

## Conclusions

The implemented experimental design declared that formula (F6) was an optimum formula, thus its composition (100 mg of the lipid core, 25 mg of PC, and 40 mg of Brij52) was involved in the fabrication of all compounds loaded EMLs. EMLs of *N*-(5-nitrothiazol-2-yl)-carboxamido derivatives (**F3a**–**g**) showed superior anti-SARS-CoV-2 activities compared to the previous unformulated ones. The IC_50_ values were recorded to be 2.87 (**F3a**), 1.51 (**F3d**), 0.73 (**F3e**), 6.22 (**F3f**), and 1.56 μg/mL (**F3g**). Obviously, the formulae of compounds **3d**, **3e**, and **3g** showed superior IC_50_ values indicating their potential as promising anti-SARS-CoV-2 drug delivery panels. Besides, the mode of action for the most potent formula (**F3e**) showed that it exhibited a combination of virucidal (>90%), viral adsorption (>80%), and viral replication (>60%) inhibition. Furthermore, molecular docking studies clarified that the most active candidates (**3d**, **3e**, and **3g**) kept the interaction with the crucial amino acid of the SARS-CoV-2 Mpro target receptor (GLU_166). Also, the histogram analysis clarified the great importance of GLU_166 in the binding pocket of SARS-CoV-2 Mpro. Therefore, we can justify the recommended antagonistic effect of the studied compounds, which explains their superior anti-SARS-CoV-2 biological effects. Furthermore, the ligand RMSD described that both **3d** and **3e** candidates exhibited stable behaviours within the binding pocket of SARS-CoV-2 Mpro during the 200 ns of the simulation time. Moreover, the MM-GBSA calculations showed that both **3d** and **3e** achieved superior Δ*G* binding energies (–56.97 and −58.73 kcal/mol, respectively) compared to that of the **Co** inhibitor (–55.25 kcal/mol). Finally, the SAR analysis clarified that increasing the size of α-substituent to the carboxamide boosts both anti-SARS-CoV-2 activity and selectivity with benzyl substitution being optimum (compound **3e**). Finally, compound **3e**-loaded EMLs (**F3e**) could be proposed as a reliable system with boosted anti-SARS-CoV-2 activity.

## Supplementary Material

Supplemental Material
