# Supplementary material for: Design and statistical optimisation of emulsomal nanoparticles for improved anti-SARS-CoV-2 activity of N-(5-nitrothiazol-2-yl)-carboxamido candidates: in vitro and in silico studies
Source: J Enzyme Inhib Med Chem. 2023 Apr 24;38(1):2202357. doi: 10.1080/14756366.2023.2202357 (PMC10128464; doi:10.1080/14756366.2023.2202357)
Supplement: Supplemental Material [file IENZ_A_2202357_SM6265.pdf]

Design and Statistical Optimization of Emulsomal Nanoparticles for Improved Anti-SARS-CoV-2 Activity of *N*-(5-Nitrothiazol-2-yl)-carboxamido Candidates: *In Vitro* and *In Silico* Studies

Ahmed A. Al-Karmalawy[a]*, Dalia S. El-Gamil[a], Rabeh EL-Shesheny[b], Marwa Sharaky[c], Radwan Alnajjar[d,e,f], Omnia Kutkat[b], Yassmin Moatasim[b], Mohamed Elagawany[g], Sara T. Al-Rashood[h], Faizah A. Binjubair[h], Wagdy M. Eldehna[i,j], Ayman M. Noreddin[k,l], and Mohamed Y. Zakaria[m]

[a] Pharmaceutical Chemistry Department, Faculty of Pharmacy, Ahram Canadian University, 6th of October City, Giza 12566, Egypt.
[b] The Center of Scientific Excellence for Influenza Viruses, Water Pollution Research Department, Environmental Research Institute, National Research Centre, Dokki-Giza 12622, Egypt.
[c] Cancer Biology Department, Pharmacology Unit, National Cancer Institute (NCI), Cairo University, Cairo, Egypt.

[d] Department of Chemistry, Faculty of Science, University of Benghazi, Benghazi, Libya.

[e] PharmD, Faculty of Pharmacy, Libyan International Medical University, Benghazi, Libya.

[f] Department of Chemistry, University of Cape Town, Rondebosch 7701, South Africa.

[g] Department of Pharmaceutical Chemistry, Faculty of Pharmacy, Damanhour University, Damanhour, Egypt.
[h] Department of Pharmaceutical Chemistry, College of Pharmacy, King Saud University, Riyadh 11451, Saudi Arabia.
[i] Department of Pharmaceutical Chemistry, Faculty of Pharmacy, Kafrelsheikh University, Kafrelsheikh, P.O. Box 33516, Egypt.
[j] School of Biotechnology, Badr University in Cairo, Badr City 11829, Egypt.
[k] Department of Internal Medicine, School of Medicine, University of California -Irvine, USA.
[l] Department of Clinical Pharmacy, Faculty of Pharmacy, Ahram Canadian University, 6th of October City, Giza 12566, Egypt.
[m] Department of Pharmaceutics and Industrial Pharmacy, Faculty of Pharmacy, Port Said University, Port Said 42526, Egypt.

* Corresponding author:

Ahmed A. Al-Karmalawy: Email: akarmalawy@acu.edu.eg

ORCID: 0000-0002-8173-6073

Supplementary Material

Materials and Methods

S1: MTT cytotoxicity assay (CC_{50})

Samples were diluted with Dulbecco's Modified Eagle's Medium (DMEM). Stock solutions of the test compounds were prepared in 10 % DMSO in *dd* H_2O. The cytotoxic activity of the extracts was tested in Vero-E6 cells by using the 3-(4, 5-dimethylthiazol-2-yl)-2, 5-diphenyltetrazolium bromide (MTT) method with minor modification [1]. Briefly, the cells were seeded in 96 well-plates (100 µL/well at a density of 3×10^5 cells/mL) and incubated for 24 h at 37 °C in 5% CO_2. After 24 h, cells were treated with various concentrations of the tested samples in triplicates. After a further 72 h, the supernatant was discarded and cell monolayers were washed with 1X sterile phosphate buffer saline (PBS) 3 times and MTT solution (20 µL of 5 mg/mL stock solution) was added to each well and incubated at 37 °C for 4 h followed by medium aspiration. In each well, the formed formazan crystals were dissolved with 200 µL of DMSO (0.04 M HCl in absolute isopropanol = 0.073 mL HCl in 50 mL isopropanol). The absorbance of formazan solutions was measured at λmax 540 nm with 620 nm as a reference wavelength using a multi-well plate reader. The plot of % cytotoxicity versus sample concentration was used to calculate the concentration which exhibited 50% cytotoxicity (TC_{50}). The % of cytotoxicity compared to the untreated cells was determined with the following equation:

$$\%\ cytotoxicity = \frac{(absorbance\ of\ cells\ without\ treatment - absorbance\ of\ cells\ with\ treatment) X 100}{absorbance\ of\ cells\ without\ treatment}$$

S2: Inhibitory concentration 50 (IC_{50}) determination

The Vero-E6 cells (2.4×104) were kept overnight at 37°C in 5% CO_2 inside 96-well tissue culture plates. 1x PBS solution was used to wash the cell monolayers for only one time which were then treated with different serial dilutions of the examined compounds together with a fixed dilution from the virus (*h*CoV-19/Egypt/NRC-03/2020 (Accession Number on GSAID: EPI_ISL_430820)) following $TCID_{50}$ test and kept at RT for 1 h before starting incubation. Also, the cell monolayers were subjected to DMEM (100 µl) with different concentrations of the test samples and virus and left at 37°C for 72 h in a 5% CO_2. Then, 4% paraformaldehyde (100 µl) was used for cell fixation (2 h) followed by the staining step with 0.1% crystal violet in distilled H_2O (50 µl) at RT for 15 min. Absolute CH_3OH (100 µl) was added to

dissolve the crystal violet dye per well to measure the optical density of the produced color using Anthos Zenyth 200rt plate reader at 570 nm.[2] The IC_{50} value for each tested compound which is corresponding to its minimum concentration required to reduce the virus infectivity by 50% in comparison to the virus control was calculated.

S3: Mode of action of virus inhibition

The possible mode of action of virus inhibition by the selected plants' extracts was examined at three different stages of the virus propagation cycle and based on three main possible modes of action: (i) Inhibition of budding and viral replication. (ii) The ability of each extract to inhibit of attachment of the virus to infected cells-membrane fusion known as blocking the viral entry (viral adsorption); and (iii) The direct effect of each extract to inactivate the virus viability (virucidal activity). Additionally, the above-mentioned mode of action could account for the recorded antiviral activities either independently, or in combinations. In this regard, the interaction between the selected plants' extracts and the MERS-CoV virus could be explained through the following three different modes of action:

S3.1. Virucidal

The virucidal assay was carried out [3] in a 6 wells plate where Vero-E6 cells were cultivated (10^5 cells/mL) for 24 h at 37 ^oC. A volume of 200 µL serum-free DMEM containing virus was added to the concentration of the tested extract. After 1 h incubation, the mixture was diluted using serum-free medium 3 times each 10-fold which still allows the existence of viral particles to grow on Vero-E6 cells but leaves nearly no extract and 100 µL of each dilution was added to the Vero-E6 cell monolayer. After 1 h contact time, DMEM over layer was added to cell monolayer. Plates were left to solidify and then incubated at 37 ^oC to allow the formation of viral plaques, fixed and stained as above mentioned to calculate percentage reduction in plaques formation in comparison to control wells where cells were infected with the virus that was not pretreated with the tested extract.

S3.2. Viral adsorption

Vero-E6 cells were cultivated in a 6 wells plate (10^5 cells/mL) for 24 h at 37 ^oC for the viral adsorption assay using the Zhang *et al.* method [4]. The plant extract was applied at different concentrations in a 200 µL medium without supplements and co-incubated with the cells for 2 h at 4 ^oC. The unabsorbed extract was removed by washing cells 3 successive times with supplements free-medium then virus diluted was co-incubated with the pretreated cells for 1 h followed by adding 3 mL DMEM supplemented with

2% agarose. Plates were left to solidify and then incubated at 37 ^{o}C to allow the formation of viral plaques, fixed and stained as above mentioned to calculate percentage reduction in plaques formation in comparison to control wells where untreated Vero-E6 cells were directly infected with the virus.

S3.3. Viral replication

The viral replication assay was carried out according to Kuo *et al.* [5] in a 6-well plate where VERO-E6 cells were cultivated (10^5 cells/mL) for 24 h at 37 ^{o}C. The virus was diluted to 10^3 PFU/well and applied directly to the cells and incubated for 1 h at 37 ^{o}C. Unabsorbed viral particles were removed by washing cells 3 successive times with supplements free-medium. The extract was applied at different concentrations, after 1 h contact time, add 3 mL of 2X DMEM medium supplemented with 2% agarose to the cell monolayer. Plates were left to solidify and incubated at 37 ^{o}C till the appearance of viral plaques. Cell monolayers were fixed in 10% formalin solution for 2 h, and stained with crystal violet. Control wells were included where Vero-E6 cells were incubated with the virus and didn't treat with the extract. Finally, plaques were counted and percentage reduction in plaques formation in comparison to control wells was recorded as above mentioned.

S4: Molecular dynamics simulations

The MD simulations were carried out using Desmond simulation package of Schrödinger LLC.[6] The NPT ensemble with the temperature 300 K and a pressure 1 bar was applied in all runs. The simulation length was 200 ns with a relaxation time 1 ps for the ligands. The OPLS3 force field parameters were used in all simulations.[7] The cutoff radius in Coulomb interactions was 9.0 Å. The orthorhombic periodic box boundaries were set 10 Å away from the protein atoms. The water molecules were explicitly described using the transferable intermolecular potential with three points (TIP3P) model.[8, 9] Salt concentration set to 0.15 M NaCl and was built using the System Builder utility of Desmond.[10] The Martyna−Tuckerman−Klein chain coupling scheme with a coupling constant of 2.0 ps was used for the pressure control and the Nosé−Hoover chain coupling scheme for the temperature control.[11, 12] Nonbonded forces were calculated using a RESPA integrator where the short-range forces were updated every step and the long-range forces were updated every three steps. The trajectories were saved at 20 ns intervals for analysis. The behavior and interactions between the ligands and protein were analyzed using the Simulation Interaction Diagram tool implemented in Desmond MD package. The stability of MD simulations was monitored by looking on the RMSD of the ligand and protein atom positions in time.

S5: MD trajectory analysis and prime MM-GBSA calculations

Simulation interactions diagram panel of Maestro software was used to monitoring interactions contribution in the ligand-protein stability. The molecular mechanics generalized born/solvent accessibility (MM – GBSA) was performed to calculate the ligand binding free energies and ligand strain energies for docked compounds over the last 50 ns with thermal_mmgbsa.py python script provided by Schrodinger which takes a Desmond trajectory file, splits it into individual snapshots, runs the MM-GBSA calculations on each frame, and outputs the average computed binding energy.

Tables

Table S1: CC_{50} and IC_{50} values of compounds formulae (**F3a-g**) against SARS-CoV-2 in Vero E6 cells *via* MTT assay [*h*CoV-19/Egypt/NRC-03/2020 (Accession Number on GSAID: EPI_ISL_430820)].

Formula	CC_{50} (µg/mL)	IC_{50} (µg/mL)	Selectivity index
**F3a**	14.29	2.87	4.97
**F3b**	57.33	$IC_{50} > CC_{50}$	-
**F3c**	19.71	$IC_{50} > CC_{50}$	-
**F3d**	8.48	1.51	5.61
**F3e**	13.40	0.73	18.35
**F3f**	37.84	6.22	6.08
**F3g**	58.98	1.56	37.80

Figures

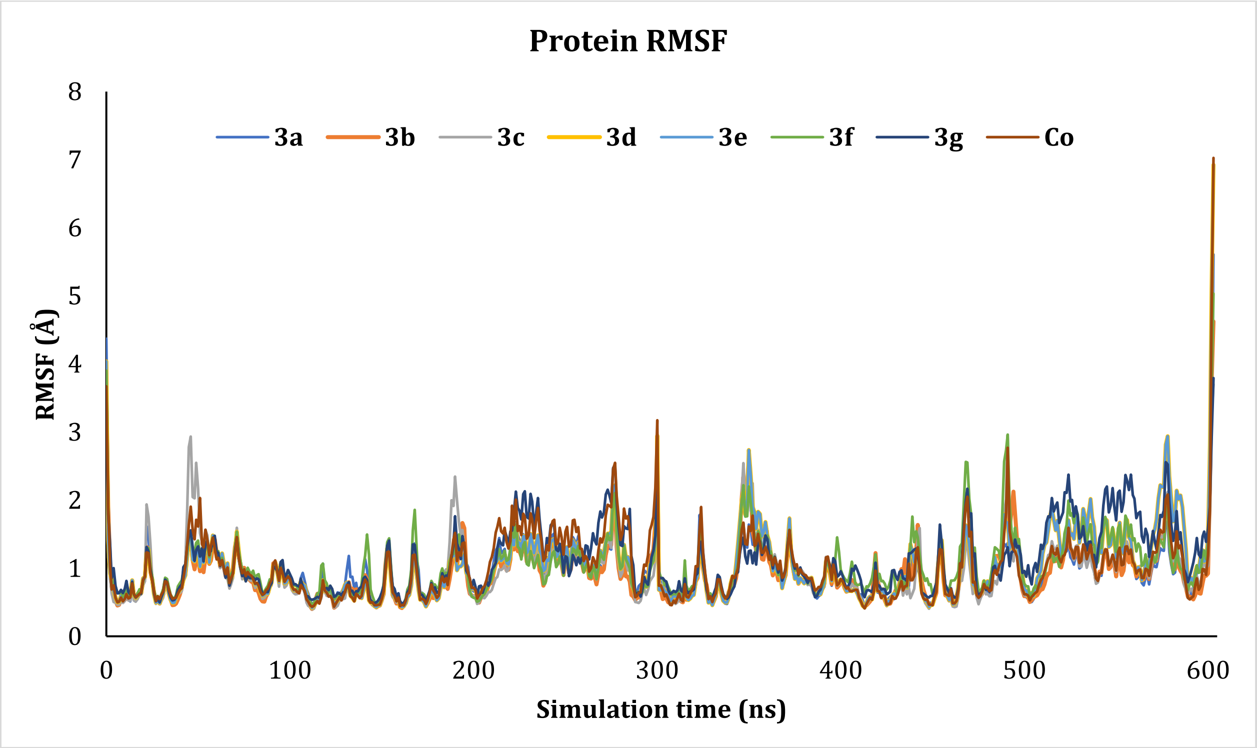

Figure S1: The RMSF of the proteins Cα within the binding pocket of SARS-CoV-2 Mpro (PDB ID: 6Y2G) during the simaultion time for compounds **3a**, **3b**, **3c**, **3d**, **3e**, **3f**, **3g**, and **Co**.

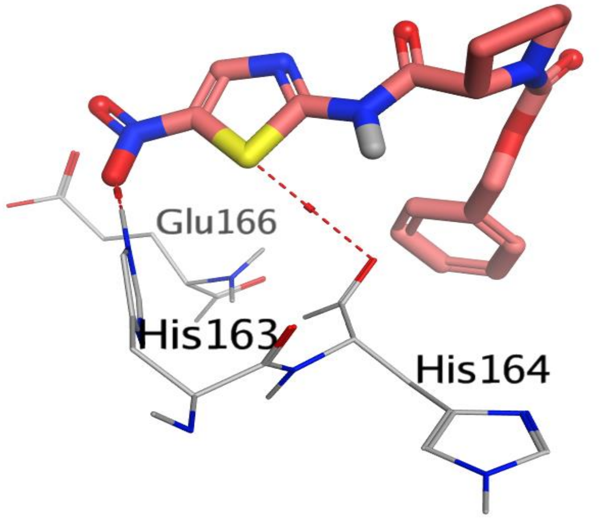

Figure S2: 3D binding interaction of compound **3f** within the binding pocket of SARS-CoV-2 Mpro (PDB ID: 6Y2G).

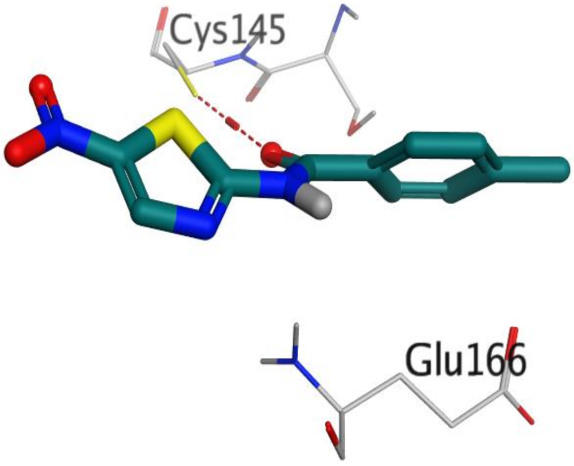

Figure S3: 3D binding interaction of compound **3a** within the binding pocket of SARS-CoV-2 Mpro (PDB ID: 6Y2G).

References

1. Alnajjar, R.; Mostafa, A.; Kandeil, A.; Al-Karmalawy, A. A., Molecular docking, molecular dynamics, and in vitro studies reveal the potential of angiotensin II receptor blockers to inhibit the COVID-19 main protease. *Heliyon* **2020,** *6* (12), e05641.
2. Marques, N. P.; Lopes, C. S.; Marques, N. C. T.; Cosme-Silva, L.; Oliveira, T. M.; Duque, C.; Sakai, V. T.; Hanemann, J. A. C., A preliminary comparison between the effects of red and infrared laser irradiation on viability and proliferation of SHED. *Lasers in medical science* **2019,** *34* (3), 465-471.
3. Schuhmacher, A.; Reichling, J.; Schnitzler, P., Virucidal effect of peppermint oil on the enveloped viruses herpes simplex virus type 1 and type 2 in vitro. *Phytomedicine : international journal of phytotherapy and phytopharmacology* **2003,** *10* (6-7), 504-10.
4. Harcourt, J.; Tamin, A.; Lu, X.; Kamili, S.; Sakthivel, S. K.; Murray, J.; Queen, K.; Tao, Y.; Paden, C. R.; Zhang, J., Severe acute respiratory syndrome coronavirus 2 from patient with coronavirus disease, United States. *Emerging infectious diseases* **2020,** *26* (6), 1266.
5. Kuo, Y. C.; Lin, L. C.; Tsai, W. J.; Chou, C. J.; Kung, S. H.; Ho, Y. H., Samarangenin B from Limonium sinense suppresses herpes simplex virus type 1 replication in Vero cells by regulation of viral macromolecular synthesis. *Antimicrobial agents and chemotherapy* **2002,** *46* (9), 2854-64.
6. Release, S., 3: Desmond molecular dynamics system, DE Shaw research, New York, NY, 2017. *Maestro-Desmond Interoperability Tools, Schrödinger, New York, NY* **2017**.
7. Harder, E.; Damm, W.; Maple, J.; Wu, C.; Reboul, M.; Xiang, J. Y.; Wang, L.; Lupyan, D.; Dahlgren, M. K.; Knight, J. L., OPLS3: a force field providing broad coverage of drug-like small molecules and proteins. *Journal of chemical theory and computation* **2016,** *12* (1), 281-296.
8. Jorgensen, W. L.; Chandrasekhar, J.; Madura, J. D.; Impey, R. W.; Klein, M. L., Comparison of simple potential functions for simulating liquid water. *The Journal of chemical physics* **1983,** *79* (2), 926-935.
9. Neria, E.; Fischer, S.; Karplus, M., Simulation of activation free energies in molecular systems. *The Journal of chemical physics* **1996,** *105* (5), 1902-1921.
10. Manual, D. U., Desmond2. 2. **2009**.
11. Martyna, G. J.; Klein, M. L.; Tuckerman, M., Nosé–Hoover chains: The canonical ensemble via continuous dynamics. *The Journal of chemical physics* **1992,** *97* (4), 2635-2643.
12. Martyna, G. J.; Tobias, D. J.; Klein, M. L., Constant pressure molecular dynamics algorithms. *The Journal of chemical physics* **1994,** *101* (5), 4177-4189.
